# Supplementary material for: The prevalence of mild-to-moderate distress in patients with end-stage renal disease: results from a patient survey using the emotion thermometers in four hospital Trusts in the West Midlands, UK
Source: BMJ Open. 2019 May 15;9(5):e027982. doi: 10.1136/bmjopen-2018-027982 (PMC6530384; doi:10.1136/bmjopen-2018-027982)
Supplement: Author's manuscript [file bmjopen-2018-027982.draft_revisions.pdf]

BMJ Open is committed to open peer review. As part of this commitment we make the peer review history of every article we publish publicly available.

When an article is published we post the peer reviewers' comments and the authors' responses online. We also post the versions of the paper that were used during peer review. These are the versions that the peer review comments apply to.

The versions of the paper that follow are the versions that were submitted during the peer review process. They are not the versions of record or the final published versions. They should not be cited or distributed as the published version of this manuscript.

BMJ Open is an open access journal and the full, final, typeset and author-corrected version of record of the manuscript is available on our site with no access controls, subscription charges or pay-per-view fees (http://bmjopen.bmj.com).

If you have any questions on BMJ Open's open peer review process please email info.bmjopen@bmj.com

BMJ Open

The prevalence of mild to moderate distress in patients with end stage renal disease: results from a multi-centre patient survey using the emotion thermometers

Journal:	*BMJ Open*
Manuscript ID	bmjopen-2018-027982
Article Type:	Research
Date Submitted by the Author:	16-Nov-2018

Complete List of Authors:	Damery, Sarah; University of Birmingham, Institute of Applied Health Research<br>Brown, Celia; University of Warwick<br>Sein, Kim; University of Birmingham, Institute of Applied Health Research<br>Nicholas, Johann; Shrewsbury and Telford Hospital NHS Trust, Renal Unit<br>Baharani, Jyoti; Heart of England NHS Foundation Trust, Renal Medicine<br>Combes, Gill; University of Birmingham, Institute of Applied Health Research

Keywords:	survey, Chronic renal failure < NEPHROLOGY, distress, End stage renal failure < NEPHROLOGY, emotion thermometers

SCHOLARONE™
Manuscripts

TITLE

The prevalence of mild to moderate distress in patients with end stage renal disease: results from a multi-centre patient survey using the emotion thermometers

AUTHORS

Sarah Damery,* Research Fellow, Institute of Applied Health Research, University of Birmingham, Edgbaston, West Midlands, B15 2TT, United Kingdom. Email: s.l.damery@bham.ac.uk; Tel: +44 (0)121 414 3343 [Corresponding author]

Celia Taylor, Associate Professor, Warwick Medical School, University of Warwick, Coventry, CV4 7AL, United Kingdom. Email: celia.taylor@warwick.ac.uk

Kim Sein, Research Fellow, Institute of Applied Health Research, University of Birmingham, Edgbaston, West Midlands, B15 2TT, United Kingdom. Email: k.sein@bham.ac.uk

Johann Nicholas, Consultant Nephrologist, Renal Unit, New Cross Hospital, Royal Wolverhampton NHS Trust, Wolverhampton Road, Wolverhampton, WV10 0QP, United Kingdom. Email: johann.nicholas@nhs.net

Jyoti Baharani, Consultant Nephrologist, Renal Unit, Birmingham Heartlands Hospital, Heart of England NHS Foundation Trust, Bordesley Green East, Birmingham, B9 5SS, United Kingdom. Email: jyoti.baharani@heartofengland.nhs.uk

Gill Combes, Senior Research Fellow, Institute of Applied Health Research, University of Birmingham, Edgbaston, West Midlands, B15 2TT, United Kingdom. Email: g.combes@bham.ac.uk

For peer review only

ABSTRACT

Objectives

To assess the prevalence of mild to moderate distress in patients with end stage renal disease and determine the association between distress and patient characteristics.

Design

Cross-sectional survey using the emotion thermometers and distress thermometer problem list.

Setting

Renal units in four hospital Trusts in the West Midlands, UK.

Participants

Adult patients with stage 5 chronic kidney disease who were: i) pre-renal replacement therapy; ii) on dialysis for less than 2 years; iii) on dialysis for 2 or more years, or iv) with a functioning transplant.

Outcomes

The prevalence of mild to moderate distress, and the incidence of distress thermometer problems and patient support needs.

Results

One third of survey respondents met the criteria for mild to moderate distress (n=346; 33.3%). Prevalence was highest in patients on dialysis for two or more years (n=109/300; 36.3%) and lowest in transplant patients (n=118/404; 29.2%). Prevalence was significantly higher in younger vs. older patients (X^2=14.33; p=0.0008), in females vs. males (X^2=6.63; p=0.01) and in black and minority ethnic patients vs. patients of white ethnicity (X^2=10.36; p=0.013). Over forty per cent of patients (n=141) reported needing support. More than 95% of patients reported physical problems and 91.9% reported at least one emotional problem.

Conclusions

Mild to moderate distress is common in patients with end stage renal disease, and there may be substantial unmet support needs. Regular screening could help identify patients whose distress may otherwise remain undetected. Further research into differences in distress prevalence over time and at specific transitional points across the renal disease pathway is needed, as is work to determine how best to support patients requiring help.

KEY WORDS

Chronic Kidney Disease, Distress, End Stage Renal Disease, emotion thermometers, survey

For peer review only

STRENGTHS AND LIMITATIONS OF THIS STUDY

- This study is the first to explore the prevalence of mild to moderate distress in a large cohort of patients with end stage renal disease (ESRD)
- Findings suggest that distress affects around a third of ESRD patients at any one time, and that there are substantial unmet support needs in this population
- The inclusion of multiple study sites is likely to have minimised any bias arising from variations in the organisation and delivery of renal services
- The survey response rate was low, and younger patients, those from black and minority ethnic (BME) groups and patients who had been more recently diagnosed were under-represented in responses
- Future research using a cohort study design would aid understanding of the ways that individuals adapt to ESRD diagnosis and its management over time.

For peer review only

For peer review only - http://bmjopen.bmj.com/site/about/guidelines.xhtml

BACKGROUND

In 2016, there were 51,672 patients with end stage renal disease (ESRD) receiving renal replacement therapy (RRT) in England.[1] Treatment is life-sustaining but not curative, and patients must constantly adjust to frequent changes to health status and likelihood of survival. Consequently, patients can experience many emotional and psychological stressors, including acceptance of diagnosis, disease progression, choosing treatment options, coping with dialysis, and associated impacts on employment, relationships and lifestyle.[2,3] Evidence suggests that patients with ESRD experience rates of depression and anxiety markedly higher than the general population.[4,5] Establishing prevalence is challenging, partly because many uraemic symptoms can be misinterpreted as symptoms of depression,[6] and partly due to the variation in prevalence estimates obtained using different diagnostic tools and modes of assessment (e.g. self-reported vs. interview-based scales).[7] Consequently, depression and anxiety prevalence estimates reported in the ESRD literature range from around 6% to 71%,[8] but are generally considered to be between 20 to 30% for dialysis patients[9] and around 25% for transplant patients.[10] This compares to a point prevalence of depression of between 2 and 9% and lifetime depression risk of around 7% in the general population.[11]

Untreated anxiety and depression in ESRD patients are associated with decreased health-related quality of life and higher symptom burden.[12] These factors may raise the risk of poor outcomes, increased healthcare use, and sub-optimal adherence with diet and medication regimes.[13-15] There is also evidence that depression status is associated with an elevated risk of all-cause mortality in renal patients, with meta-analysis suggesting an excess mortality risk attributable to depression higher than that observed in other chronic diseases such as cancer and diabetes.[7] Provision of emotional and psychological support is central to the recommended management of renal disease, and both the United Kingdom Department of Health and NICE (National Institute for Health and Care Excellence) mandate provision of such support within national renal guidelines.[16,17]

Whilst supportive services are relatively advanced for those with higher level needs requiring psychiatric or psychological intervention, there is a lack of robust data on the prevalence of lower-level support needs – defined as difficulties adjusting to, and coping effectively with, renal failure, diagnosis, physical symptoms and treatment. It may also be useful to broaden the focus beyond narrowly-defined anxiety and depression to consider the determinants and consequences of more general emotional and psychological difficulty encompassed by the term 'distress'. Whilst linear progression from lower-level to severe difficulty is not inevitable, timely identification of patient distress may facilitate effective management. This study aimed to assess the prevalence of mild to moderate distress in a cohort of ESRD patients and determine the association between distress and sociodemographic and clinical characteristics. It forms part of a larger mixed methods study that aims to understand how the recognition and management of patients' emotional and psychological support needs can be integrated into the ESRD pathway.[18]

METHODS

The study used a cross-sectional survey, distributed by post to all eligible ESRD patients managed at four National Health Service (NHS) hospital Trusts in the West Midlands, UK. Trusts were chosen to maximise diversity in patient demographics, catchment size, urban-rural mix and the organisation of psychological support services: site 1 (small) and site 2 (large) both serve urban, inner city catchments with substantial

For peer review only - http://bmjopen.bmj.com/site/about/guidelines.xhtml

black and minority ethnic (BME) populations. Site 3 (medium) and site 4 (large) both serve predominantly white populations in urban areas with surrounding rural districts. Sites 2 and 4 have access to a renal psychologist for the provision of psychological support services, whereas sites 1 and 3 do not.

Participants and recruitment

Eligible patients were aged 18+ years, diagnosed with chronic kidney disease (CKD) stage 5, and grouped according to their stage on the ESRD pathway: i) pre-RRT, ii) on peritoneal or haemodialysis for less than two years, iii) on dialysis for two or more years, or iv) with a functioning transplant. Although differentiating according to dialysis vintage is not a recognised clinical distinction, it was hypothesised by the clinicians involved in designing this study that there may be differences in distress prevalence between patients initiating dialysis more recently vs. those undergoing dialysis for longer. Patients using psychiatric services since CKD5 diagnosis were excluded. Renal unit staff at each Trust identified eligible patients from hospital records, and survey packs were prepared on Trust premises by the University research team. Eligible patients received a letter of invitation from the renal unit's lead consultant, an information sheet and a survey, to be returned directly to the researchers. Return envelopes were marked with a unique identifier for recording returns, and non-responders received one reminder after six weeks. Mailings were carried out between January 2016 and May 2017.

Survey

Although previous studies have typically used validated tools designed to measure anxiety and depression such as the Hospital Anxiety and Depression Score (HADS), this survey measured distress using the Emotion Thermometers (ET),[19,20] which allow the broader measurement of emotional and psychological difficulty considered more appropriate for this study. The ET use a visual analogue scale covering five domains: distress, anxiety, depression, anger and perceived need for help. Patients score each domain on an 11-point Likert scale to rate their levels of emotional upset during the preceding week, where '0' corresponds to none and '10' denotes extreme problems. In combining multiple domains, the ET has greater sensitivity than the distress thermometer (DT) alone in identifying emotional difficulty and broadly defined distress.[19] Alongside the ET, the survey included the Distress Thermometer Problem List[21] which has been validated in the UK renal population and lists 36 problems across five domains: practical (n=5), family (n=3), emotional (n=6), spiritual (n=1) and physical (n=21). Patients indicated which (if any) of the 36 problems they had experienced in the previous week. Closed questions covered sociodemographic characteristics (age, gender, ethnicity) and treatment modality (where relevant).

Data analysis

Thresholds for distress using the ET have been validated,[19,20] with a score of 4 to 5 denoting mild distress, and 6 to 7 denoting moderate distress. Patients were defined as having mild to moderate distress if they: a) scored between 4 to 7 on the DT (regardless of scores in the other ET domains), or b) scored 0 to 3 on the DT and 4 to 7 on one or more of the anxiety, depression and anger thermometers, with no thermometer exceeding 7. Analysis was descriptive, focusing on associations between distress and respondents' stage on the ESRD pathway and sociodemographic characteristics. Anonymised data were obtained from hospital information systems to allow a comparison of the characteristics of respondents and non-respondents on the basis of age group, gender, ethnic group and ESRD pathway stage. Comparisons were undertaken using

For peer review only - http://bmjopen.bmj.com/site/about/guidelines.xhtml

chi-squared analysis. Any surveys in which the emotion thermometers were left blank by a respondent were excluded from analysis. The prevalence of total and individual problems from the DT problem list were analysed descriptively, and medians and inter-quartile ranges were calculated to compare numbers of problems cited within each domain. Data were analysed using SPSS (version 21.0, Armonk, NY: IBM Corp).

Sample size

The primary outcome was the difference in the prevalence of mild to moderate distress across patients at different ESRD stages. We anticipated an average prevalence of 25% across all patients,[9] with patients in the stages with the highest and lowest prevalence at +/- 5 percentage points from this average (i.e. 20% for the stage with the lowest prevalence and 30% for the stage with the highest prevalence). This equated to a small effect size (w) of approximately 0.1. To detect this difference with 80% power and 5% significance, a total of 1090 responses were required (assuming approximately equal numbers of patients in each ESRD stage).

Ethical approval

Ethics approval was obtained from the Coventry and Warwickshire Research Ethics Committee in October 2015 [Ref 15/WM/0288]. The study was also approved by the Research Governance office of each of the participating hospital Trusts.

RESULTS

A total of 3730 surveys were sent across the four study sites. One hundred patients died between the initial and reminder mailings (2.7%) and 2442 recipients (65.5%) did not respond. Of the 1188 responses received (31.8%), 148 were removed due to non-completion of the emotion thermometers (4.0%), giving a total of 1040 valid responses (27.9%) (Figure 1). Rates of valid responses ranged from 23.0% in site 1 to 30.4% in site 4. Younger patients (<65 years old) were significantly less likely to respond than those aged 65 and over, as were those from BME groups compared to those in the white ethnic group. Patients yet to begin RRT and those on dialysis for less than 2 years were significantly less likely to respond than patients with a transplant or who had been undergoing dialysis for 2 or more years. There was no significant difference between responders and non-responders on the basis of gender (Table 1).

Characteristics of respondents

The majority of respondents were male (n=633; 60.9%) and in the white ethnic group (n=902; 86.7%) (Table 2). Patients aged between 51 and 69 years old constituted the largest age group (n=441; 42.9%), with those aged under 50 comprising 16.9% of the total (n=174). Nearly two fifths of respondents had received a transplant (n=404; 38.8%) and 28.8% had been on dialysis for 2 or more years (n=300). Of the 454 patients undergoing regular dialysis, the most common modality was hospital/in-centre haemodialysis (n=343; 75.6%).

Prevalence of distress

A total of 561 respondents (53.9%) were categorised as having none to low distress, and a further 133 patients (12.3%) met the criteria for severe distress (i.e. scoring greater than 7 on one or more of the

For peer review only - http://bmjopen.bmj.com/site/about/guidelines.xhtml

emotion thermometers). A total of 346 respondents (33.3%) met the criteria for mild to moderate distress (hereafter termed 'distress'). Distress prevalence by hospital site was lowest in site 4 (30.9%) and highest in site 1 (38.5%), although the difference between study sites was not statistically significant (X^2 = 3.24; p=0.356). Distress was identified in 208 patients (60.1%) on the basis of their DT score alone, with the remaining 138 (39.9%) identified from their scores on the anxiety, depression or anger thermometers. Distress prevalence was highest in patients who had been on dialysis for 2 or more years (n=109/300; 36.3%) and lowest in transplant patients (n=118/404; 29.2%). For dialysis modality, distress was most prevalent for home haemodialysis patients (n=13/31; 41.9%) and lowest in the peritoneal dialysis group (n=22/80; 27.5%), although numbers were small. There was no significant difference in rates of distress on the basis of ESRD stage or dialysis type when groups were compared. In contrast, all sociodemographic characteristics showed significant differences in distress prevalence between groups. Patients in the youngest age group were significantly more likely to report distress than those in the older age groups (n=78/174; 44.8%; X^2 = 14.33; p=0.0008). The prevalence of distress was significantly higher for females than males (38.1% vs. 30.2%; X^2 = 6.63; p=0.01) and for BME patients compared to white patients (45.7% vs. 31.4%; X^2 = 10.36; p=0.0013).

Perceived need for help

Scores on the 'need' thermometer were assessed as an outcome measure indicating patients' perceived need for help. Scores of four and above were considered to indicate unmet support needs[19] (Table 3). Patients at study sites 1 and 3 had significantly higher perceived support needs than those in sites 2 and 4 (29.4% and 22.9% vs. 21.5% and 20.1%; X^2 = 9.49; p=0.02). There were also significant differences by sub-group according to ESRD stage: 66.0% of patients on dialysis for less than two years (n=35) required support, as did 55.7% of patients on dialysis for two or more years (n=59). Perceived support needs in the pre-RRT and transplant groups were significantly lower, at 34.9% and 29.9% respectively (X^2 = 27.71; p<0.0001). Support needs were also significantly higher in BME compared to white patients, at 57.6% vs. 37.9% (X^2 = 7.06; p=0.008).

Distress thermometer problem list

All but six patients reported experiencing at least one problem on the DT problem list in the preceding week (98.0%) (Table 4). The most frequently reported problems were all in the emotional and physical domains, with 91.9% of patients reporting at least one emotional problem in the previous week, and 95.1% reporting at least one physical problem. 'Worry' was the commonest problem across all domains (n=247; 74.3%). 'Fatigue' (71.7%), 'dry/itchy skin' (65.0%) and 'sleep' (61.3%) were the most common physical problems. 'Spiritual problems' (6.6%) and 'problems with childcare' (family domain: 3.8%) were reported least frequently. The median number of total problems reported was 12/36 (IQR: 7 to 16) (Table 5).

Sub-group analysis shows that transplant patients reported significantly fewer physical problems and total problems than pre-RRT or dialysis patients (median physical problems 6/21, IQR 3 to 10, p=0.019; median total problems 10/36, IQR 5 to 16, p=0.023). Patients aged 70 and older reported significantly fewer problems on the practical, family and emotional domains than younger patients. They also reported significantly fewer total problems. Females reported significantly more physical problems than males (median 8/21, IQR 5 to 12 vs. 7/21, IQR 5 to 11; p=0.043).

For peer review only - http://bmjopen.bmj.com/site/about/guidelines.xhtml

DISCUSSION

Detecting distress is important for the optimal care of patients with ESRD, and evidence suggests that reducing emotional and psychological difficulties may enhance wellbeing and improve patients' ability to engage with complex and demanding treatments. Our study has shown that as many as one in three patients with ESRD may have mild to moderate distress, and rates of reporting emotional and physical problems were high. This was evident across the ESRD pathway, with around 35% of pre-RRT and dialysis patients and 29% of transplant patients meeting the criteria for mild to moderate distress. The finding that the prevalence of distress was almost as high in transplant patients as those undergoing dialysis suggests that although they may experience fewer physical problems, the need for ongoing psychological adjustment does not end at the point of transplantation.[10] Mild to moderate distress was most prevalent in the group of patients who had been on dialysis for 2 or more years (36.3%), which may reflect a lack of adjustment and coping in this group with the ongoing challenges of undergoing regular dialysis treatment over an extended period, potentially declining health, and the limitations that dialysis treatment places on family, relationships and lifestyle. There was also variation by dialysis type, with patients receiving hospital/in-centre haemodialysis reporting distress rates of 37.6%, compared to 27.5% for patients undergoing peritoneal dialysis. Numbers were too small to detect statistically significant differences, but this distinction has been found elsewhere,[22] and would benefit from further study.

Distress prevalence was strongly associated with sociodemographic characteristics and was significantly higher in younger vs. older patients, in BME vs. white patients, and in females compared to males. These trends have also been found in studies of anxiety and depression in renal patients.[23,24] There are numerous psychological theories of health and illness which may have a role in explaining patients' variable responses to ESRD diagnosis and treatment.[25-27] These theories emphasise that experience of distress is likely to be determined by an individual's personal degree of resilience rather than being associated with specific clinical characteristics, so our observed association between sociodemographic characteristics and distress may confirm the influence of individual coping resources on distress.

Comparable national figures for the pre-RRT group are not available, but if the prevalence of mild to moderate distress found in this study was standardised to the current population in England with a functioning transplant or undergoing dialysis, it would equate to 18,970 ESRD patients experiencing difficulties, of whom 7,835 may want help.[1] Identifying and managing distress is challenging and the method, targeting and frequency of assessment should be carefully considered. Experiencing distress may also be considered a normal response to chronic disease diagnosis, and it has been argued that the primary goal of supportive services should be to distinguish between transient and ongoing difficulties and focus on optimising patients' ability to cope with their condition by managing their distress.[28] In this context, it is likely that the most feasible interventions are those that can be incorporated into routine clinical practice and standard protocols for ESRD care. The ET could be used regularly by clinicians during patient consultations or by renal nurses to screen for distress. Screening in itself will not necessarily lead to better patient outcomes, but it may help to identify patients whose distress would otherwise remain undetected, and in doing so, could enable the targeted provision of support services.

For peer review only - http://bmjopen.bmj.com/site/about/guidelines.xhtml

It is also important to recognise that not all patients with mild to moderate distress will want to receive support. Although we found that the likelihood of renal patients experiencing symptoms of distress is predominantly associated with age, gender and ethnicity, the complementary finding that dialysis patients were particularly likely to report a perceived need for support suggests that targeting screening for distress towards those who undergo regular hospital or in-centre haemodialysis may be beneficial. Here, screening would seem particularly important both for younger patients, who may experience significantly greater disruption to their family and working life than older patients, and for patients in BME groups who reported uniformly high rates of mild to moderate distress and a correspondingly pronounced perceived need for support. Providing appropriate information to patients early on in the ESRD pathway (pre-RRT) about the distress they may experience could also help to manage patients' expectations and allow timely identification of emotional and psychological difficulties. There was also evidence of a centre effect in the proportion of patients reporting a perceived need for support at participating study sites. Patients in sites 1 and 3, where there is no access to a renal psychologist reported significantly higher rates of support need than those in sites 2 and 4 where renal psychologist support is available. This suggests that the availability of in-house psychological support services may play an important role in helping ESRD patients to manage distress.

Limitations

The survey response rate was low, and younger patients, those from BME groups and patients who had been more recently diagnosed were under-represented in responses. The study was marginally underpowered, with 1040 valid responses received, rather than the 1090 required by our *a priori* sample size estimation. We are also unable to say whether distress is more or less common in survey respondents compared to non-respondents, nor could we perform detailed sub-group analyses for patients within each ESRD pathway stage. This meant that participants in each ESRD pathway stage were treated as a homogeneous cohort when in reality there may have been differences between them that may have impacted on their experience of distress such as issues with medication or ESRD-related complications like fistula failure, infection or transplant failure. However, in-depth qualitative research was undertaken with renal patients to explore the detailed determinants and consequences of their distress in a linked component of this study (findings to be reported elsewhere).

Nevertheless, our findings with regard to the patient groups most likely to experience distress were similar to existing evidence on anxiety and depression in renal disease. Because younger, BME patients were under-represented in our sample, the finding that patients in these groups reported significantly higher rates of mild to moderate distress than older, white patients, suggests that we may have under-estimated rather than over-estimated overall distress prevalence. The inclusion of multiple study sites is likely to have minimised any bias that may arise from variations in the organisation and delivery of renal services. Patients referred to psychiatric services (as noted in their hospital record) were excluded, but we cannot know whether our sample included patients who had independently sought counselling or support via their general practitioner. The study was also cross-sectional rather than longitudinal, and consequently allows limited understanding of the relationship between time since ESRD diagnosis and ability to cope with the resulting stressors. Some studies with renal patients have found no correlation between depressive symptoms and time since treatment initiation,[9] whereas others have found a tendency for depression status to worsen.[29] Future work

For peer review only - http://bmjopen.bmj.com/site/about/guidelines.xhtml

using a cohort study design would aid understanding of the ways that individuals adapt to ESRD diagnosis and its ongoing management over time.

CONCLUSION

This study is, to our knowledge, the first to explore the prevalence of mild to moderate distress in a large cohort of ESRD patients. Our findings show that mild to moderate distress is common – even after transplantation – and there may be substantial unmet patient support needs. Further research into possible variations in distress prevalence over time and at different transitional points across the ESRD pathway is needed, as is work to determine how best to support patients requiring help.

For peer review only

DECLARATIONS

Funding

This research was funded by the United Kingdom National Institute for Health Research (NIHR) Collaboration for Leadership in Applied Health Research and Care West Midlands (CLAHRCWM). The views expressed in this manuscript are those of the authors and not necessarily those of the National Health Service, the NIHR or the Department of Health and Social Care.The funder had no role in study design, the collection, analysis and interpretation of data, or in writing the manuscript.

Competing interests

All authors declare that they have no competing interests.

Author contributions

SD managed the survey, undertook all statistical analysis and drafted the first version of the manuscript. CT contributed to research design and helped to develop the methodological/analytical approach. KS contributed to data analysis, interpretation and refinement of study methodology. JN and JB both participated in research design and in the development and refinement of study methodology. GC conceived the study, was chief investigator, and contributed to all aspects of research design and methodology. All authors have seen and approved the final version of the manuscript.

Data sharing

No additional data are available.

For peer review only

REFERENCES

1. Byrne C, Caskey F, Castledine C, Dawnay A, Ford D, Fraser S et al. 19th Annual Report of the Renal Association. *NEPHRON* 2017; **137** Suppl 1. UK Renal Registry: Bristol, UK.

2. Gregory DM, Way CY, Hutchinson TA, Barrett BJ, Parfrey PS. Patients' perceptions of their experiences with ESRD and hemodialysis treatment. *Qual Health Res* 1998; **8(6)**: 764-83.

3. Kimmel PL, Peterson RA. Depression in end-stage renal disease patients treated with hemodialysis: tools, correlates, outcomes, and needs. *Semin Dial* 2005; **18(2)**: 91-7.

4. Zalai D, Szeifert L, Novak M. Psychological distress and depression in patients with chronic kidney disease. *Semin Dial* 2012; **25(4)**: 428-38.

5. Hedayati SS, Yalamanchili V, Finkelstein FO. A practical approach to the treatment of depression in patients with chronic kidney disease and end-stage renal disease. *Kidney Int* 2012; **81(3)** :247-55.

6. McKercher C, Sanderson K, Jose MD. Psychosocial factors in people with chronic kidney disease prior to renal replacement therapy. *Nephrology* 2013; **18(9)**: 585-91.

7. Palmer S, Vecchio M, Craig JC, Tonelli M, Johnson DW, Nicolucci A et al. Prevalence of depression in chronic kidney disease: systematic review and meta-analysis of observational studies. *Kidney Int* 2013; **84(1)**: 179-91.

8. Chilcot J, Wellsted D, Da Silva-Gane M, Farrington K. Depression on dialysis. *Nephron Clin Pract* 2008; **108(4)**: 256-64.

9. Cukor D. Use of CBT to treat depression among patients on hemodialysis. *Psychiatr Serv* 2007; **58(5)**: 711-12.

10. Chilcot J, Spencer BW, Maple H, Mamode N. Depression and kidney transplantation. *Transplantation* 2014; **97(7)**: 717-21.

For peer review only - http://bmjopen.bmj.com/site/about/guidelines.xhtml

11. Cohen SD, Norris L, Acquaviva K, Peterson RA, Kimmel PL. Screening, diagnosis and treatment of depression in patients with end stage renal disease. *Clin J Am Soc Nephrol* 2007; **2(6)**: 1332-42.

12. Soni RK, Weisbord SD, Unruh ML. Health-related quality of life outcomes in chronic kidney disease. *Curr Opin Nephrol Hypertens* 2010; **19(2)**: 153-9.

13. Cukor D, Rosenthal DS, Jindal RM, Brown CD, Kimmel PL. Depression is an important contributor to low medication adherence in hemodialyzed patients and transplant recipients. *Kidney Int* 2009; **75(11)**: 1223-9.

14. Clark S, Farrington K, Chilcot J. Nonadherence in dialysis patients: prevalence, measurement, outcome, and psychological determinants. *Semin Dial* 2014; **27(1)**: 42–9.

15. Weisbord SD, Fried LF, Mor MK, Resnick AL, Unruh ML, Palevsky PM et al. Renal provider recognition of symptoms in patients on maintenance hemodialysis. *Clin J Am Soc Nephrol* 2007; **2(5)**: 960-7.

16. Department of Health. The National Service Framework for Renal Services. Part two: chronic kidney disease, acute renal failure and end of life care. London: Department of Health, 2005.

17. National Institute for Health and Care Excellence. GS5 Chronic Kidney Disease quality standard. London: NICE, 2011.

18. Taylor F, Taylor C, Baharani J, Nicholas J, Combes G. Integrating emotional and psychological support into the end-stage renal disease pathway: a protocol for mixed methods research to identify patients' lower-level support needs and how these can most effectively be addressed. *BMC Nephrol* 2016; **17**: 111.

19. Mitchell AJ, Baker-Glenn EA, Granger L, Symonds P. Can the Distress Thermometer be improved by additional mood domains? Part I. Initial validation of the Emotion Thermometers tool. *Psychooncology* 2010; **19(2)**: 125–33.

20. Mitchell AJ, Baker-Glenn EA, Granger L, Symonds P. Can the Distress Thermometer be improved by additional mood domains? Part II. What is the optimal combination of Emotion Thermometers? *Psychooncology* 2010; **19(2)**: 134–40.

For peer review only

For peer review only - http://bmjopen.bmj.com/site/about/guidelines.xhtml

21. Holland JC, Bultz BD. The NCCN guideline for distress management: a case for making distress the sixth vital sign. *J Natl Compr Canc Netw* 2007; **5(1)**: 3-7.

22. Kalender B, Ozdemir C, Dervisoglu E, Ozdemir O. Quality of life in chronic kidney disease: effects of treatment modality, depression, malnutrition and inflammation. *Int J Clin Pract* 2007; **61(4)**: 569-76.

23. Drayer RA, Piraino B, Reynolds CF III, Houck PR, Mazumder S, Bernardini J et al. Characteristics of depression in hemodialysis patients: symptoms, quality of life and mortality risk. *Gen Hosp Psychiatry* 2006; **28(4)**: 306–12.

24. Harwood L, Wilson B, Locking-Cusolito H, Sontrop J, Spittal J. Stressors and coping in individuals with chronic kidney disease. *Nephrol Nurs J* 2009; **36(3)**: 265-76.

25. Cvengros JA, Christensen AJ, Lawton WJ. Health locus of control and depression in chronic kidney disease: a dynamic perspective. *J Health Psychol* 2005; **10(5)**: 677-86.

26. Birmele B, Le Gall A, Sautenet B, Aguerre C, Camus V. Clinical, sociodemographic, and psychological correlates of health-related quality of life in chronic hemodialysis patients. *Psychosomatics* 2012; **53(1)**: 30-7.

27. Chilcot J, Wellsted D, Davenport A, Farrington K. Illness representations and concurrent depression symptoms in haemodialysis patients. *J Health Psychol* 2011; **16(7)**: 1127-37.

28. Moss-Morris R. Adjusting to chronic illness: time for a unified theory. *Br J Health Psychol* 2013; **18(4)**: 681–6.

29. Lopes AA, Bragg J, Young E, Goodkin D, Mapes D, Combe C et al. Depression as a predictor of mortality and hospitalization among hemodialysis patients in the United States and Europe. *Kidney Int* 2002; **62(1)**: 199-207.

For peer review only - http://bmjopen.bmj.com/site/about/guidelines.xhtml

FIGURE LEGEND

Figure 1: Surveys mailed and returned

For peer review only

For peer review only - http://bmjopen.bmj.com/site/about/guidelines.xhtml

TABLES

Table 1: Comparison of the characteristics of survey responders and non-responders

Characteristic	Respondents	Non-respondents	Total	Comparison of proportions
Stage on ESRD pathway				
Pre-RRT	183	560	743	
Dialysis <2 years	162	481	643	**X^2 = 9.96; p=0.02**
Dialysis 2+ years	293	684	977	
Transplant	402	965	1367	
Age group†				
Less 65	503	1537	2040	**X^2 = 19.86; p<0.0001**
65 and above	524	1151	1675	
Gender				
Male	635	1636	2271	X^2 = 0.01; p=0.92
Female	405	1054	1459	
Ethnicity‡				
White	902	1720	2622	**X^2 = 76.16; p<0.0001**
Black and minority ethnic	138	635	773	

† Age unknown for 15 patients; ‡ Ethnicity unknown for 335 patients; bold text indicates statistical significance

For peer review only

Table 2: Respondent characteristics and proportion by sub-group with mild to moderate distress

Characteristic	Respondents (%)	Mild to moderate distress (%)	Comparison of proportions (mild to moderate distress patients)†
All respondents	1040 (100.0)	346 (33.3)	
Study site			
Site 1	187 (18.0)	72 (38.5)	
Site 2	177 (17.0)	58 (32.8)	$X^2 = 3.24$; p=0.356
Site 3	323 (31.1)	107 (33.1)	
Site 4	353 (33.9)	109 (30.9)	
Stage on ESRD pathway			
Pre-RRT	182 (17.5)	64 (35.2)	
Dialysis <2 years	154 (14.8)	55 (35.7)	$X^2 = 4.85$; p=0.183
Dialysis 2+ years	300 (28.8)	109 (36.3)	
Transplant	404 (38.8)	118 (29.2)	
Dialysis type (n=454)			
Hospital/in-centre haemodialysis	343 (75.6)	129 (37.6)	
Home haemodialysis	31 (6.8)	13 (41.9)	$X^2 = 3.36$; p=0.186
Peritoneal dialysis	80 (17.6)	22 (27.5)	
Age group‡			
Less than 50	174 (16.9)	78 (44.8)	
50 to 69	441 (42.9)	145 (32.9)	**$X^2 = 14.33$; p=0.0008**
70 and above	414 (40.2)	119 (28.7)	
Gender			
Male	633 (60.9)	191 (30.2)	**$X^2 = 6.63$; p=0.01**
Female	407 (39.1)	155 (38.1)	
Ethnicity			
White	902 (86.7)	283 (31.4)	**$X^2 = 10.36$; p=0.0013**
Black and minority ethnic	138 (13.3)	63 (45.7)	

†Bold text denotes a statistically significant difference between groups; ‡Age unknown for four respondents

For peer review only

For peer review only - http://bmjopen.bmj.com/site/about/guidelines.xhtml

Table 3: Perceived need for help with distress by sub-group with mild to moderate distress (n=346)

Characteristic	Median 'need' score (IQR)	Patients scoring 4 or more on need thermometer (%)	Comparison of proportions (mild to moderate distress patients)†
All respondents	3 (1 to 5)	141 (40.8)	
Study site			
Site 1	1 (0 to 4)	55 (29.4)	
Site 2	1 (0 to 3)	38 (21.5)	**X^2 = 9.49; p=0.02**
Site 3	1 (0 to 3)	74 (22.9)	
Site 4	0 (0 to 3)	71 (20.1)	
Stage on ESRD pathway			
Pre-RRT	2 (0 to 4)	22 (34.9)	
Dialysis <2 years	3 (2 to 6)	35 (66.0)	**X^2 = 27.71; p<0.0001**
Dialysis 2+ years	4 (2 to 6)	59 (55.7)	
Transplant	2 (0 to 4)	35 (29.9)	
Dialysis type (n=454)			
Hospital/in-centre haemodialysis	4 (2 to 6)	70 (56.0)	
Home haemodialysis	4 (2 to 8)	7 (53.8)	X^2 = 5.44; p=0.07
Peritoneal dialysis	3 (0 to 5)	6 (28.6)	
Age group			
Less than 50	3 (2 to 5)	35 (44.9)	
50 to 69	3 (1 to 5)	55 (39.0)	X^2 = 0.85; p=0.654
70 and above	3 (1 to 5)	48 (41.4)	
Gender			
Male	3 (1 to 5)	73 (39.2)	X^2 = 0.54; p=0.462
Female	3 (1 to 5)	67 (43.8)	
Ethnicity			
White	3 (1 to 5)	106 (37.9)	**X^2 = 7.06; p=0.008**
Black and minority ethnic	5 (2 to 7)	34 (57.6)	

†Bold text indicates a statistically significant difference between groups

For peer review only

Table 4: Number and proportion of patients reporting problems on the DT problem list

**Problems by domain**	**Number of patients (%)†**	**Patients reporting at least 1 problem for the domain (%)**
Any problems (n=36)	-	339 (98.0)
Practical domain (n=5)		
Transport	84 (24.3)	
Insurance/financial	76 (22.0)	
Work	53 (15.3)	167 (48.3)
Housing	30 (8.7)	
Childcare	13 (3.8)	
Family domain (n=3)		
Dealing with friend/relative	66 (19.1)	
Dealing with partner	60 (17.3)	118 (34.1)
Dealing with children	27 (7.8)	
Emotional domain (n=6)		
Worry	257 (74.3)	
Loss of interest in usual activities	186 (53.8)	
Sadness	166 (48.0)	318 (91.9)
Depression	159 (46.0)	
Nervousness	150 (43.4)	
Fears	148 (42.8)	
Spiritual domain (n=1)		
Spiritual/religious concerns	23 (6.6)	-
Physical domain (n=21)		
Fatigue	248 (71.7)	
Skin dry/itchy	225 (65.0)	
Sleep	212 (61.3)	
Memory/concentration	183 (52.9)	
Pain	178 (51.4)	
Getting around	171 (49.4)	
Tingling in hands/feet	144 (41.6)	
Breathing	133 (38.4)	
Feeling swollen	127 (36.7)	
Bathing/dressing	119 (34.4)	329 (95.1)
Appearance	116 (33.5)	
Eating	111 (32.1)	
Nose dry/congested	108 (31.2)	
Constipation	106 (30.6)	
Nausea	100 (28.9)	
Changes in urination	94 (27.2)	
Indigestion	92 (26.6)	
Sexual	88 (25.4)	
Diarrhoea	79 (22.8)	
Mouth sores	42 (12.1)	
Fevers	31 (9.0)	

†Problems within each domain ranked from most to least frequently cited by respondents

For peer review only

For peer review only - http://bmjopen.bmj.com/site/about/guidelines.xhtml

Table 5: Median number of problems reported in each DT problem list domain, by patient group

**Characteristic**	**Practical[†,‡]**	**Family**	**Emotional**	**Physical**	**Total**
Stage on ESRD pathway	Median (IQR)[§]	Median (IQR)	Median (IQR)	Median (IQR)	Median (IQR)
Pre-RRT	1 (0 to 1)	0 (0 to 1)	3 (1 to 4)	**8 (4 to 12)**	**12 (7 to 17)**
Dialysis <2 years	1 (0 to 2)	0 (0 to 1)	3 (2 to 5)	**8 (6 to 12)**	**13 (9 to 17)**
Dialysis 2+ years	1 (0 to 1)	0 (0 to 1)	3 (2 to 5)	**9 (6 to 11)**	**13 (9 to 16)**
Transplant	0 (0 to 1)	0 (0 to 1)	3 (1 to 4)	**6 (3 to 10)**	**10 (5 to 16)**
				**p=0.019**	**p=0.023**
Dialysis type (n=164)					
Hospital/in-centre haemodialysis	1 (0 to 1)	0 (0 to 1)	3 (2 to 5)	8 (5 to 11)	13 (8 to 16)
Home haemodialysis	1 (0 to 2)	0 (0 to 1)	4 (2 to 5)	10 (8 to 13)	16 (11 to 19)
Peritoneal dialysis	0 (0 to 1)	0 (0 to 1)	3 (2 to 4)	8 (6 to 11)	12 (9 to 16)
Age group[4]					
Less than 50	**1 (0 to 2)**	**0 (0 to 1)**	**3 (2 to 5)**	7 (4 to 11)	**13 (8 to 17)**
50 to 69	**1 (0 to 2)**	**0 (0 to 1)**	**3 (2 to 5)**	8 (5 to 11)	**12 (8 to 18)**
70 and above	**0 (0 to 1)**	**0 (0 to 0)**	**3 (1 to 4)**	7 (4 to 10)	**11 (7 to 15)**
	**p<0.0001**	**p=0.001**	**p=0.038**		**p=0.033**
Gender					
Male	1 (0 to 1)	0 (0 to 1)	3 (2 to 4)	**7 (4 to 10)**	12 (7 to 16)
Female	0 (0 to 1)	0 (0 to 1)	3 (2 to 5)	**8 (5 to 12)**	12 (8 to 17)
				**p=0.043**	
Ethnicity					
White	0 (0 to 1)	0 (0 to 1)	3 (2 to 4)	8 (5 to 11)	12 (7 to 16)
Black and minority ethnic	0 (0 to 2)	0 (0 to 1)	4 (1 to 5)	7 (4 to 10)	11 (7 to 17)
All respondents (n=346)	0 (0 to 1)	0 (0 to 1)	3 (2 to 5)	8 (5 to 11)	12 (7 to 16)

[†]Bold text denotes a statistically significant difference between groups (Mann Whitney test for characteristics with two sub-groups; Kruskall Wallis test for characteristics with three or more sub-groups); [‡]Spiritual domain excluded from analysis; [§]IQR = inter-quartile range

For peer review only

For peer review only - http://bmjopen.bmj.com/site/about/guidelines.xhtml

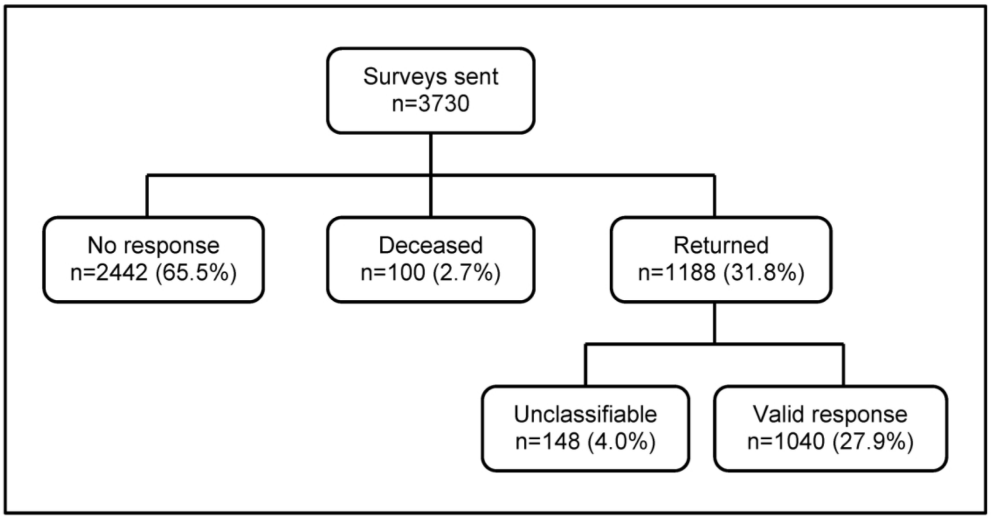

Figure 1: Surveys mailed and returned

150x78mm (300 x 300 DPI)

STROBE 2007 (v4) Statement—Checklist of items that should be included in reports of *cross-sectional studies*

Section/Topic	Item #	Recommendation	Reported on page #
**Title and abstract**	1	(*a*) Indicate the study's design with a commonly used term in the title or the abstract	1
		(*b*) Provide in the abstract an informative and balanced summary of what was done and what was found	2
**Introduction**			
Background/rationale	2	Explain the scientific background and rationale for the investigation being reported	4
Objectives	3	State specific objectives, including any prespecified hypotheses	4
**Methods**			
Study design	4	Present key elements of study design early in the paper	5-6
Setting	5	Describe the setting, locations, and relevant dates, including periods of recruitment, exposure, follow-up, and data collection	4-5
Participants	6	(*a*) Give the eligibility criteria, and the sources and methods of selection of participants	5
Variables	7	Clearly define all outcomes, exposures, predictors, potential confounders, and effect modifiers. Give diagnostic criteria, if applicable	5-6
Data sources/ measurement	8*	For each variable of interest, give sources of data and details of methods of assessment (measurement). Describe comparability of assessment methods if there is more than one group	5-6
Bias	9	Describe any efforts to address potential sources of bias	*5*
Study size	10	Explain how the study size was arrived at	6
Quantitative variables	11	Explain how quantitative variables were handled in the analyses. If applicable, describe which groupings were chosen and why	5-6
Statistical methods	12	(*a*) Describe all statistical methods, including those used to control for confounding	5-6
		(*b*) Describe any methods used to examine subgroups and interactions	5-6
		(*c*) Explain how missing data were addressed	6
		(*d*) If applicable, describe analytical methods taking account of sampling strategy	N/A
		(*e*) Describe any sensitivity analyses	N/A
**Results**			

For peer review only

Participants	13*	(a) Report numbers of individuals at each stage of study—eg numbers potentially eligible, examined for eligibility, confirmed eligible, included in the study, completing follow-up, and analysed	6; Fig 1
		(b) Give reasons for non-participation at each stage	N/A
		(c) Consider use of a flow diagram	Fig 1
Descriptive data	14*	(a) Give characteristics of study participants (eg demographic, clinical, social) and information on exposures and potential confounders	6
		(b) Indicate number of participants with missing data for each variable of interest	N/A
Outcome data	15*	Report numbers of outcome events or summary measures	6-7
Main results	16	(*a*) Give unadjusted estimates and, if applicable, confounder-adjusted estimates and their precision (eg, 95% confidence interval). Make clear which confounders were adjusted for and why they were included	6-7
		(*b*) Report category boundaries when continuous variables were categorized	6-7
		(*c*) If relevant, consider translating estimates of relative risk into absolute risk for a meaningful time period	N/A
Other analyses	17	Report other analyses done—eg analyses of subgroups and interactions, and sensitivity analyses	6-7
**Discussion**			
Key results	18	Summarise key results with reference to study objectives	8
Limitations	19	Discuss limitations of the study, taking into account sources of potential bias or imprecision. Discuss both direction and magnitude of any potential bias	9
Interpretation	20	Give a cautious overall interpretation of results considering objectives, limitations, multiplicity of analyses, results from similar studies, and other relevant evidence	8
Generalisability	21	Discuss the generalisability (external validity) of the study results	9-10
**Other information**			
Funding	22	Give the source of funding and the role of the funders for the present study and, if applicable, for the original study on which the present article is based	11

*Give information separately for cases and controls in case-control studies and, if applicable, for exposed and unexposed groups in cohort and cross-sectional studies.

Note: An Explanation and Elaboration article discusses each checklist item and gives methodological background and published examples of transparent reporting. The STROBE checklist is best used in conjunction with this article (freely available on the Web sites of PLoS Medicine at http://www.plosmedicine.org/, Annals of Internal Medicine at http://www.annals.org/, and Epidemiology at http://www.epidem.com/). Information on the STROBE Initiative is available at www.strobe-statement.org.

For peer review only

The prevalence of mild to moderate distress in patients with end stage renal disease: results from a patient survey using the emotion thermometers in four hospital Trusts in the West Midlands, UK

Journal:	*BMJ Open*
Manuscript ID	bmjopen-2018-027982.R1
Article Type:	Research
Date Submitted by the Author:	14-Mar-2019
Complete List of Authors:	Damery, Sarah; University of Birmingham, Institute of Applied Health Research<br>Brown, Celia ; University of Warwick<br>Sein, Kim; University of Birmingham, Institute of Applied Health Research<br>Nicholas, Johann; Shrewsbury and Telford Hospital NHS Trust, Renal Unit<br>Baharani, Jyoti; Heart of England NHS Foundation Trust, Renal Medicine<br>Combes, Gill; University of Birmingham, Institute of Applied Health Research
**Primary Subject Heading**:	Renal medicine
Secondary Subject Heading:	Epidemiology, Communication, Health services research
Keywords:	survey, Chronic renal failure < NEPHROLOGY, distress, End stage renal failure < NEPHROLOGY, emotion thermometers

TITLE

The prevalence of mild to moderate distress in patients with end stage renal disease: results from a patient survey using the emotion thermometers in four hospital Trusts in the West Midlands, UK.

AUTHORS

Sarah Damery,* Research Fellow, Institute of Applied Health Research, University of Birmingham, Edgbaston, West Midlands, B15 2TT, United Kingdom. Email: s.l.damery@bham.ac.uk; Tel: +44 (0)121 414 3343 [Corresponding author]

Celia Brown,[§] Associate Professor, Warwick Medical School, University of Warwick, Coventry, CV4 7AL, United Kingdom. Email: celia.taylor@warwick.ac.uk

Kim Sein, Research Fellow, Institute of Applied Health Research, University of Birmingham, Edgbaston, West Midlands, B15 2TT, United Kingdom. Email: k.sein@bham.ac.uk

Johann Nicholas, Consultant Nephrologist, Renal Unit, Shrewsbury and Telford NHS Trust, Mytton Oak Road, Shrewsbury, SY3 8XQ, United Kingdom. Email: johann.nicholas@nhs.net

Jyoti Baharani, Consultant Nephrologist, Renal Unit, Birmingham Heartlands Hospital, Heart of England NHS Foundation Trust, Bordesley Green East, Birmingham, B9 5SS, United Kingdom. Email: jyoti.baharani@heartofengland.nhs.uk

Gill Combes, Senior Research Fellow, Institute of Applied Health Research, University of Birmingham, Edgbaston, West Midlands, B15 2TT, United Kingdom. Email: g.combes@bham.ac.uk

[§] Celia Taylor at the time the research was undertaken

For peer review only

ABSTRACT

Objectives

To assess the prevalence of mild to moderate distress in patients with end stage renal disease and determine the association between distress and patient characteristics.

Design

Cross-sectional survey using the emotion thermometers and distress thermometer problem list.

Setting

Renal units in four hospital Trusts in the West Midlands, UK.

Participants

Adult patients with stage 5 chronic kidney disease who were: i) pre-renal replacement therapy; ii) on dialysis for less than 2 years; iii) on dialysis for 2 or more years, or iv) with a functioning transplant.

Outcomes

The prevalence of mild to moderate distress, and the incidence of distress thermometer problems and patient support needs.

Results

One third of survey respondents met the criteria for mild to moderate distress (n=346; 33.3%). Prevalence was highest in patients on dialysis for two or more years (n=109/300; 36.3%) and lowest in transplant patients (n=118/404; 29.2%). Prevalence was significantly higher in younger vs. older patients (X^2=14.33; p=0.0008), in females vs. males (X^2=6.63; p=0.01) and in black and minority ethnic patients vs. patients of white ethnicity (X^2=10.36; p=0.013). Over forty per cent of patients (n=141) reported needing support. More than 95% of patients reported physical problems and 91.9% reported at least one emotional problem.

Conclusions

Mild to moderate distress is common in patients with end stage renal disease, and there may be substantial unmet support needs. Regular screening could help identify patients whose distress may otherwise remain undetected. Further research into differences in distress prevalence over time and at specific transitional points across the renal disease pathway is needed, as is work to determine how best to support patients requiring help.

KEY WORDS

Chronic Kidney Disease, Distress, End Stage Renal Disease, emotion thermometers, survey

For peer review only

STRENGTHS AND LIMITATIONS OF THIS STUDY

- This study is the first to explore the prevalence of mild to moderate distress in a large cohort of patients with end stage renal disease (ESRD)
- Findings suggest that distress affects around a third of patients with ESRD at any one time, and that there are substantial unmet support needs in this population
- The inclusion of multiple study sites is likely to have minimised any bias arising from variations in the organisation and delivery of renal services
- The survey response rate was low, and younger patients, those from black and minority ethnic (BME) groups and patients who had been more recently diagnosed were under-represented in responses
- Future research using a cohort study design would aid understanding of the ways that individuals adapt to ESRD diagnosis and its management over time.

For peer review only

BACKGROUND

In 2016, there were 51,672 patients with end stage renal disease (ESRD) receiving renal replacement therapy (RRT) in England.[1] Treatment is life-sustaining but not curative, and patients must constantly adjust to frequent changes to health status and likelihood of survival. Consequently, patients can experience many emotional and psychological stressors, including acceptance of diagnosis, disease progression, choosing treatment options, coping with dialysis, and associated impacts on employment, relationships and lifestyle.[2,3] Evidence suggests that patients with ESRD experience rates of depression and anxiety markedly higher than the general population.[4,5] Establishing prevalence is challenging, partly because many uraemic symptoms can be misinterpreted as symptoms of depression,[6] and partly due to the variation in prevalence estimates obtained using different diagnostic tools and modes of assessment (e.g. self-reported vs. interview-based scales).[7] Consequently, depression and anxiety prevalence estimates reported in the ESRD literature range from around 6% to 71%,[8] but are generally considered to be between 20 to 30% for dialysis patients[9] and around 25% for transplant patients.[10] This compares to a point prevalence of depression of between 2 and 9% and lifetime depression risk of around 7% in the general population.[11]

Untreated anxiety and depression in patients with ESRD are associated with decreased health-related quality of life and higher symptom burden.[12] These factors may raise the risk of poor outcomes, increased healthcare use, and sub-optimal adherence with diet and medication regimes.[13-15] There is also evidence that depression status is associated with an elevated risk of all-cause mortality in renal patients, with meta-analysis suggesting an excess mortality risk attributable to depression higher than that observed in other chronic diseases such as cancer and diabetes.[7] Provision of emotional and psychological support is central to the recommended management of renal disease, and both the United Kingdom Department of Health and NICE (National Institute for Health and Care Excellence) mandate provision of such support within national renal guidelines.[16,17]

Whilst supportive services are relatively advanced for those with higher level needs requiring psychiatric or psychological intervention, there is a lack of robust data on the prevalence of lower-level support needs – defined as difficulties adjusting to, and coping effectively with, renal failure, diagnosis, physical symptoms and treatment. It may also be useful to broaden the focus beyond narrowly-defined anxiety and depression to consider the determinants and consequences of more general emotional and psychological difficulty encompassed by the term ‘distress’. Whilst linear progression from lower-level to severe difficulty is not inevitable, timely identification of patient distress may facilitate effective management. This study aimed to assess the prevalence of mild to moderate distress in a cohort of patients with ESRD and determine the association between distress and sociodemographic and clinical characteristics. It forms part of a larger mixed methods study that aims to understand how the recognition and management of patients’ emotional and psychological support needs can be integrated into the ESRD pathway.[18]

METHODS

The study used a cross-sectional survey, distributed by post to all eligible patients with ESRD managed at four National Health Service (NHS) hospital Trusts in the West Midlands, UK. Trusts were chosen to maximise diversity in patient demographics, catchment size, urban-rural mix and the organisation of psychological support services: site 1 (small) and site 2 (large) both serve urban, inner city catchments with

For peer review only - http://bmjopen.bmj.com/site/about/guidelines.xhtml

substantial black and minority ethnic (BME) populations. Site 3 (medium) and site 4 (large) both serve predominantly white populations in urban areas with surrounding rural districts. Sites 2 and 4 have access to a renal psychologist for the provision of psychological support services, whereas sites 1 and 3 do not.

Participants and recruitment

Eligible patients were aged 18+ years, diagnosed with chronic kidney disease (CKD) stage 5, and grouped according to their stage on the ESRD pathway: i) pre-RRT, ii) on peritoneal or haemodialysis for less than two years, iii) on dialysis for two or more years, or iv) with a functioning transplant. Although differentiating according to dialysis vintage is not a recognised clinical distinction, it was hypothesised by the clinicians involved in designing this study that there may be differences in distress prevalence between patients initiating dialysis more recently vs. those undergoing dialysis for longer. Patients using psychiatric services since CKD5 diagnosis were excluded. Renal unit staff at each Trust identified eligible patients from hospital records, and survey packs were prepared on Trust premises by the University research team. Eligible patients received a letter of invitation from the renal unit's lead consultant, an information sheet and a survey, to be returned directly to the researchers. Return envelopes were marked with a unique identifier for recording returns, and non-responders received one reminder after six weeks. Mailings were carried out between January 2016 and May 2017.

Survey

The survey measured distress with the Emotion Thermometers (ET),[19,20] which use a visual analogue scale covering five domains: distress, anxiety, depression, anger and perceived need for help. Patients score each domain on an 11-point Likert scale to rate their levels of emotional upset during the preceding week, where '0' corresponds to none and '10' denotes extreme problems. Although not validated specifically for use with renal patients, the ET has been widely used in studies of patients with cancer and other chronic conditions where it has been found to be sensitive in identifying emotional difficulty and broadly defined distress.[19] It incorporates the distress thermometer (DT), which has been validated in the UK population with chronic kidney disease.[21] The survey also included the Distress Thermometer Problem List[22] which lists 36 problems across five domains: practical (n=5), family (n=3), emotional (n=6), spiritual (n=1) and physical (n=21). Patients indicated which (if any) of the 36 problems they had experienced in the previous week. Closed questions covered sociodemographic characteristics (age, gender, ethnicity) and treatment modality (where relevant).

Data analysis

Thresholds for distress using the ET have been validated,[19,20] with a score of 4 to 5 denoting mild distress, and 6 to 7 denoting moderate distress. Patients were defined as having mild to moderate distress if they: a) scored between 4 to 7 on the DT (regardless of scores in the other ET domains), or b) scored 0 to 3 on the DT and 4 to 7 on one or more of the anxiety, depression and anger thermometers, with no thermometer exceeding 7. Analysis was descriptive, focusing on associations between distress and respondents' stage on the ESRD pathway and sociodemographic characteristics. Anonymised data were obtained from hospital information systems to allow a comparison of the characteristics of respondents and non-respondents on the basis of age group, gender, ethnic group and ESRD pathway stage. Comparisons were undertaken using chi-squared analysis. Any surveys in which the emotion thermometers were left blank by a respondent were

For peer review only - http://bmjopen.bmj.com/site/about/guidelines.xhtml

excluded from analysis. The prevalence of total and individual problems from the DT problem list were analysed descriptively, and medians and inter-quartile ranges were calculated to compare numbers of problems cited within each domain. Data were analysed using SPSS (version 21.0, Armonk, NY: IBM Corp).

Sample size

The primary outcome was the difference in the prevalence of mild to moderate distress across patients at different ESRD stages. We anticipated an average prevalence of 25% across all patients,[9] with patients in the stages with the highest and lowest prevalence at +/- 5 percentage points from this average (i.e. 20% for the stage with the lowest prevalence and 30% for the stage with the highest prevalence). This equated to a small effect size (w) of approximately 0.1. To detect this difference with 80% power and 5% significance, a total of 1090 responses were required (assuming approximately equal numbers of patients in each ESRD stage).

Ethical approval

Ethics approval was obtained from the Coventry and Warwickshire Research Ethics Committee in October 2015 [Ref 15/WM/0288]. The study was also approved by the Research Governance office of each of the participating hospital Trusts.

Patient and public involvement

The study design and research questions were developed with input from a study advisory group that included patient representatives, and the patient and public involvement (PPI) group attached to the chronic diseases theme of the CLAHRC West Midlands. These groups were also involved in selecting appropriate outcome measures that optimised data quality whilst minimising participant burden. Patients were not involved in recruitment to the study or its conduct. Study participants will be sent a plain English summary of final study results if they indicated during the informed consent process that they would like to receive this.

RESULTS

A total of 3730 surveys were sent across the four study sites. One hundred patients died between the initial and reminder mailings (2.7%) and 2442 recipients (65.5%) did not respond. Of the 1188 responses received (31.8%), 148 were removed due to non-completion of the emotion thermometers (4.0%), giving a total of 1040 valid responses (27.9%) (Figure 1). Rates of valid responses ranged from 23.0% in site 1 to 30.4% in site 4. Younger patients (<65 years old) were significantly less likely to respond than those aged 65 and over, as were those from BME groups compared to those in the white ethnic group. Patients yet to begin RRT and those on dialysis for less than 2 years were significantly less likely to respond than patients with a transplant or who had been undergoing dialysis for 2 or more years. There was no significant difference between responders and non-responders on the basis of gender (Table 1).

Characteristics of respondents

The majority of respondents were male (n=633; 60.9%) and in the white ethnic group (n=902; 86.7%) (Table 2). Patients aged between 51 and 69 years old constituted the largest age group (n=441; 42.9%), with those aged under 50 comprising 16.9% of the total (n=174). Nearly two fifths of respondents had received a transplant (n=404; 38.8%) and 28.8% had been on dialysis for 2 or more years (n=300). Of the 454 patients

For peer review only - http://bmjopen.bmj.com/site/about/guidelines.xhtml

undergoing regular dialysis, the most common modality was hospital/in-centre haemodialysis (n=343; 75.6%).

Prevalence of distress

A total of 561 respondents (53.9%) were categorised as having none to low distress, and a further 133 patients (12.3%) met the criteria for severe distress (i.e. scoring greater than 7 on one or more of the emotion thermometers). A total of 346 respondents (33.3%) met the criteria for mild to moderate distress (hereafter termed 'distress'). Distress prevalence by hospital site was lowest in site 4 (30.9%) and highest in site 1 (38.5%), although the difference between study sites was not statistically significant ($X^2 = 3.24$; p=0.356). Distress was identified in 208 patients (60.1%) on the basis of their DT score alone, with the remaining 138 (39.9%) identified from their scores on the anxiety, depression or anger thermometers. Distress prevalence was highest in patients who had been on dialysis for 2 or more years (n=109/300; 36.3%) and lowest in transplant patients (n=118/404; 29.2%). For dialysis modality, distress was most prevalent for home haemodialysis patients (n=13/31; 41.9%) and lowest in the peritoneal dialysis group (n=22/80; 27.5%), although numbers were small. There was no significant difference in rates of distress on the basis of ESRD stage or dialysis type when groups were compared. In contrast, all sociodemographic characteristics showed significant differences in distress prevalence between groups. Patients in the youngest age group were significantly more likely to report distress than those in the older age groups (n=78/174; 44.8%; $X^2 = 14.33$; p=0.0008). The prevalence of distress was significantly higher for females than males (38.1% vs. 30.2%; $X^2 = 6.63$; p=0.01) and for BME patients compared to white patients (45.7% vs. 31.4%; $X^2 = 10.36$; p=0.0013).

Perceived need for help

Scores on the 'need' thermometer were assessed as an outcome measure indicating patients' perceived need for help. Scores of four and above were considered to indicate unmet support needs[19] (Table 3). Patients at study sites 1 and 3 had significantly higher perceived support needs than those in sites 2 and 4 (29.4% and 22.9% vs. 21.5% and 20.1%; $X^2 = 9.49$; p=0.02). There were also significant differences by sub-group according to ESRD stage: 66.0% of patients on dialysis for less than two years (n=35) required support, as did 55.7% of patients on dialysis for two or more years (n=59). Perceived support needs in the pre-RRT and transplant groups were significantly lower, at 34.9% and 29.9% respectively ($X^2 = 27.71$; p<0.0001). Support needs were also significantly higher in BME compared to white patients, at 57.6% vs. 37.9% ($X^2 = 7.06$; p=0.008).

Distress thermometer problem list

All but six patients reported experiencing at least one problem on the DT problem list in the preceding week (98.0%) (Table 4). The most frequently reported problems were all in the emotional and physical domains, with 91.9% of patients reporting at least one emotional problem in the previous week, and 95.1% reporting at least one physical problem. 'Worry' was the commonest problem across all domains (n=247; 74.3%). 'Fatigue' (71.7%), 'dry/itchy skin' (65.0%) and 'sleep' (61.3%) were the most common physical problems. 'Spiritual problems' (6.6%) and 'problems with childcare' (family domain: 3.8%) were reported least frequently. The median number of total problems reported was 12/36 (IQR: 7 to 16) (Table 5).

For peer review only - http://bmjopen.bmj.com/site/about/guidelines.xhtml

Sub-group analysis shows that transplant patients reported significantly fewer physical problems and total problems than pre-RRT or dialysis patients (median physical problems 6/21, IQR 3 to 10, p=0.019; median total problems 10/36, IQR 5 to 16, p=0.023). Patients aged 70 and older reported significantly fewer problems on the practical, family and emotional domains than younger patients. They also reported significantly fewer total problems. Females reported significantly more physical problems than males (median 8/21, IQR 5 to 12 vs. 7/21, IQR 5 to 11; p=0.043).

DISCUSSION

Detecting distress is important for the optimal care of patients with ESRD, and evidence suggests that reducing emotional and psychological difficulties may enhance wellbeing and improve patients' ability to engage with complex and demanding treatments. Our study has shown that as many as one in three patients with ESRD may have mild to moderate distress, and rates of reporting emotional and physical problems were high. This was evident across the ESRD pathway, with around 35% of pre-RRT and dialysis patients and 29% of transplant patients meeting the criteria for mild to moderate distress. The finding that the prevalence of distress was almost as high in transplant patients as those undergoing dialysis suggests that although they may experience fewer physical problems, the need for ongoing psychological adjustment does not end at the point of transplantation.[10] Mild to moderate distress was most prevalent in the group of patients who had been on dialysis for 2 or more years (36.3%), which may reflect a lack of adjustment and coping in this group with the ongoing challenges of undergoing regular dialysis treatment over an extended period, potentially declining health, and the limitations that dialysis treatment places on family, relationships and lifestyle. There was also variation by dialysis type, with patients receiving hospital/in-centre haemodialysis reporting distress rates of 37.6%, compared to 27.5% for patients undergoing peritoneal dialysis. Numbers were too small to detect statistically significant differences, but this distinction has been found elsewhere,[23] and would benefit from further study.

Distress prevalence was strongly associated with sociodemographic characteristics and was significantly higher in younger vs. older patients, in BME vs. white patients, and in females compared to males. These trends have also been found in studies of anxiety and depression in renal patients.[24,25] There are numerous psychological theories of health and illness which may have a role in explaining patients' variable responses to ESRD diagnosis and treatment.[26-28] These theories emphasise that experience of distress is likely to be determined by an individual's personal degree of resilience and individual coping resources rather than being associated with specific clinical characteristics.

Comparable national figures for the pre-RRT group are not available, but if the prevalence of mild to moderate distress found in this study was standardised to the current population in England with a functioning transplant or undergoing dialysis, it would equate to 18,970 patients with ESRD experiencing difficulties, of whom 7,835 may want help.[1] It has been argued that the primary goal of supportive services should be to distinguish between transient and ongoing difficulties and focus on optimising patients' ability to cope with their condition.[29] In this context, it is likely that the most feasible supportive interventions are those that can be incorporated into routine clinical practice and standard protocols for ESRD care. The ET could be used regularly by clinicians during patient consultations or by renal nurses to screen for distress. Screening

For peer review only - http://bmjopen.bmj.com/site/about/guidelines.xhtml

in itself will not necessarily lead to better patient outcomes, but it may help to identify patients whose distress would otherwise remain undetected, and in doing so, could enable the targeted provision of support services.

Experiencing distress may be considered a normal response to chronic disease diagnosis, and not all patients with mild to moderate distress will want to receive support. The likelihood that patients with ESRD will experience symptoms of distress was predominantly associated with age, gender and ethnicity. Consequently, screening for distress would seem particularly important for younger patients, who may experience significantly greater disruption to their family and working life than older patients, females, and for patients in BME groups, who reported uniformly high rates of mild to moderate distress and high support needs. It may also be useful to target screening for distress towards those who undergo regular hospital or in-centre haemodialysis. Providing appropriate information to patients early on in the ESRD pathway (pre-RRT) about the distress they may experience could also help to manage patients' expectations and allow timely identification of emotional and psychological difficulties. There was also evidence of a centre effect in the proportion of patients reporting a perceived need for support at participating study sites. Patients in sites 1 and 3, where there is no access to a renal psychologist reported significantly higher rates of support need than those in sites 2 and 4 where renal psychologist support is available. This suggests that the availability of in-house psychological support services may play an important role in helping patients with ESRD to manage distress.

Limitations

The survey response rate was low, and younger patients, those from BME groups and patients who had been more recently diagnosed were under-represented in responses. The study was marginally underpowered, with 1040 valid responses received, rather than the 1090 required by our *a priori* sample size estimation. We are also unable to say whether distress is more or less common in survey respondents compared to non-respondents, nor could we perform detailed sub-group analyses for patients within each ESRD pathway stage. This meant that participants in each ESRD pathway stage were treated as a homogeneous cohort when in reality there may have been differences between them that may have impacted on their experience of distress such as issues with medication or ESRD-related complications like fistula failure, infection or transplant failure. However, in-depth qualitative research was undertaken with renal patients to explore the detailed determinants and consequences of their distress in a linked component of this study (findings to be reported elsewhere).

Nevertheless, our findings with regard to the patient groups most likely to experience distress were similar to existing evidence on anxiety and depression in renal disease. Because younger, BME patients were under-represented in our sample, the finding that patients in these groups reported significantly higher rates of mild to moderate distress than older, white patients, suggests that we may have under-estimated rather than over-estimated overall distress prevalence. The inclusion of multiple study sites is likely to have minimised any bias that may arise from variations in the organisation and delivery of renal services. Patients referred to psychiatric services (as noted in their hospital record) were excluded, but we cannot know whether our sample included patients who had independently sought counselling or support via their general practitioner. The study was also cross-sectional rather than longitudinal, and consequently allows limited understanding of the relationship between time since ESRD diagnosis and ability to cope with the resulting stressors. Some

For peer review only - http://bmjopen.bmj.com/site/about/guidelines.xhtml

studies with renal patients have found no correlation between depressive symptoms and time since treatment initiation,[9] whereas others have found a tendency for depression status to worsen.[30] Future work using a cohort study design would aid understanding of the ways that individuals adapt to ESRD diagnosis and its ongoing management over time.

CONCLUSION

This study is, to our knowledge, the first to explore the prevalence of mild to moderate distress in a large cohort of patients with ESRD. Our findings show that mild to moderate distress is common – even after transplantation – and there may be substantial unmet patient support needs. Further research into possible variations in distress prevalence over time and at different transitional points across the ESRD pathway is needed, as is work to determine how best to support patients requiring help.

For peer review only

For peer review only - http://bmjopen.bmj.com/site/about/guidelines.xhtml

DECLARATIONS

Funding

This research was funded by the United Kingdom National Institute for Health Research (NIHR) Collaboration for Leadership in Applied Health Research and Care West Midlands (CLAHRCWM). The views expressed in this manuscript are those of the authors and not necessarily those of the National Health Service, the NIHR or the Department of Health and Social Care.The funder had no role in study design, the collection, analysis and interpretation of data, or in writing the manuscript.

Competing interests

All authors declare that they have no competing interests.

Author contributions

SD managed the survey, undertook all statistical analysis and drafted the first version of the manuscript. CB contributed to research design and helped to develop the methodological/analytical approach. KS contributed to data analysis, interpretation and refinement of study methodology. JN and JB both participated in research design and in the development and refinement of study methodology. GC conceived the study, was chief investigator, and contributed to all aspects of research design and methodology. All authors have seen and approved the final version of the manuscript.

Acknowledgements

We would like to thank the renal patient advisory group and the patient and public involvement group of the chronic disease theme of CLAHRCWM for their contribution to study design and choice of data collection tools.

Exclusive licence

I, the Submitting Author has the right to grant and does grant on behalf of all authors of the Work (as defined in the below author licence), an exclusive licence and/or a non-exclusive licence for contributions from authors who are: i) UK Crown employees; ii) where BMJ has agreed a CC-BY licence shall apply, and/or iii) in accordance with the terms applicable for US Federal Government officers or employees acting as part of their official duties; on a worldwide, perpetual, irrevocable, royalty-free basis to BMJ Publishing Group Ltd ("BMJ") its licensees and where the relevant Journal is co-owned by BMJ to the co-owners of the Journal, to publish the Work in BMJ Open and any other BMJ products and to exploit all rights, as set out in our licence.

For peer review only

Data sharing

No additional data are available.

For peer review only

For peer review only - http://bmjopen.bmj.com/site/about/guidelines.xhtml

REFERENCES

1. Byrne C, Caskey F, Castledine C, Dawnay A, Ford D, Fraser S et al. 19th Annual Report of the Renal Association. *NEPHRON* 2017; **137** Suppl 1. UK Renal Registry: Bristol, UK.

2. Gregory DM, Way CY, Hutchinson TA, Barrett BJ, Parfrey PS. Patients' perceptions of their experiences with ESRD and hemodialysis treatment. *Qual Health Res* 1998; **8(6)**: 764-83.

3. Kimmel PL, Peterson RA. Depression in end-stage renal disease patients treated with hemodialysis: tools, correlates, outcomes, and needs. *Semin Dial* 2005; **18(2)**: 91-7.

4. Zalai D, Szeifert L, Novak M. Psychological distress and depression in patients with chronic kidney disease. *Semin Dial* 2012; **25(4)**: 428-38.

5. Hedayati SS, Yalamanchili V, Finkelstein FO. A practical approach to the treatment of depression in patients with chronic kidney disease and end-stage renal disease. *Kidney Int* 2012; **81(3)** :247-55.

6. McKercher C, Sanderson K, Jose MD. Psychosocial factors in people with chronic kidney disease prior to renal replacement therapy. *Nephrology* 2013; **18(9)**: 585-91.

7. Palmer S, Vecchio M, Craig JC, Tonelli M, Johnson DW, Nicolucci A et al. Prevalence of depression in chronic kidney disease: systematic review and meta-analysis of observational studies. *Kidney Int* 2013; **84(1)**: 179-91.

8. Chilcot J, Wellsted D, Da Silva-Gane M, Farrington K. Depression on dialysis. *Nephron Clin Pract* 2008; **108(4)**: 256-64.

9. Cukor D. Use of CBT to treat depression among patients on hemodialysis. *Psychiatr Serv* 2007; **58(5)**: 711-12.

10. Chilcot J, Spencer BW, Maple H, Mamode N. Depression and kidney transplantation. *Transplantation* 2014; **97(7)**: 717-21.

For peer review only - http://bmjopen.bmj.com/site/about/guidelines.xhtml

11. Cohen SD, Norris L, Acquaviva K, Peterson RA, Kimmel PL. Screening, diagnosis and treatment of depression in patients with end stage renal disease. *Clin J Am Soc Nephrol* 2007; **2(6)**: 1332-42.

12. Soni RK, Weisbord SD, Unruh ML. Health-related quality of life outcomes in chronic kidney disease. *Curr Opin Nephrol Hypertens* 2010; **19(2)**: 153-9.

13. Cukor D, Rosenthal DS, Jindal RM, Brown CD, Kimmel PL. Depression is an important contributor to low medication adherence in hemodialyzed patients and transplant recipients. *Kidney Int* 2009; **75(11)**: 1223-9.

14. Clark S, Farrington K, Chilcot J. Nonadherence in dialysis patients: prevalence, measurement, outcome, and psychological determinants. *Semin Dial* 2014; **27(1)**: 42–9.

15. Weisbord SD, Fried LF, Mor MK, Resnick AL, Unruh ML, Palevsky PM et al. Renal provider recognition of symptoms in patients on maintenance hemodialysis. *Clin J Am Soc Nephrol* 2007; **2(5)**: 960-7.

16. Department of Health. The National Service Framework for Renal Services. Part two: chronic kidney disease, acute renal failure and end of life care. London: Department of Health, 2005.

17. National Institute for Health and Care Excellence. GS5 Chronic Kidney Disease quality standard. London: NICE, 2011.

18. Taylor F, Taylor C, Baharani J, Nicholas J, Combes G. Integrating emotional and psychological support into the end-stage renal disease pathway: a protocol for mixed methods research to identify patients' lower-level support needs and how these can most effectively be addressed. *BMC Nephrol* 2016; **17**: 111.

19. Mitchell AJ, Baker-Glenn EA, Granger L, Symonds P. Can the Distress Thermometer be improved by additional mood domains? Part I. Initial validation of the Emotion Thermometers tool. *Psychooncology* 2010; **19(2)**: 125–33.

20. Mitchell AJ, Baker-Glenn EA, Granger L, Symonds P. Can the Distress Thermometer be improved by additional mood domains? Part II. What is the optimal combination of Emotion Thermometers? *Psychooncology* 2010; **19(2)**: 134–40.

For peer review only - http://bmjopen.bmj.com/site/about/guidelines.xhtml

21. Alston H, Vickerstaff V, Low J, Beaty C, Da Silva Gane M, Burns A. Validation of the distress thermomets in a UK renal population. *Nephrol Dial Transplant* 2015; **30(Supplement 3)**: iii612.

22. Holland JC, Bultz BD. The NCCN guideline for distress management: a case for making distress the sixth vital sign. *J Natl Compr Canc* Netw 2007; **5(1)**: 3-7.

23. Kalender B, Ozdemir C, Dervisoglu E, Ozdemir O. Quality of life in chronic kidney disease: effects of treatment modality, depression, malnutrition and inflammation. *Int J Clin Pract* 2007; **61(4)**: 569-76.

24. Drayer RA, Piraino B, Reynolds CF III, Houck PR, Mazumder S, Bernardini J et al. Characteristics of depression in hemodialysis patients: symptoms, quality of life and mortality risk. *Gen Hosp Psychiatry* 2006; **28(4)**: 306–12.

25. Harwood L, Wilson B, Locking-Cusolito H, Sontrop J, Spittal J. Stressors and coping in individuals with chronic kidney disease. *Nephrol Nurs J* 2009; **36(3)**: 265-76.

26. Cvengros JA, Christensen AJ, Lawton WJ. Health locus of control and depression in chronic kidney disease: a dynamic perspective. *J Health Psychol* 2005; **10(5)**: 677-86.

27. Birmele B, Le Gall A, Sautenet B, Aguerre C, Camus V. Clinical, sociodemographic, and psychological correlates of health-related quality of life in chronic hemodialysis patients. *Psychosomatics* 2012; **53(1)**: 30-7.

28. Chilcot J, Wellsted D, Davenport A, Farrington K. Illness representations and concurrent depression symptoms in haemodialysis patients. *J Health Psychol* 2011; **16(7)**: 1127-37.

29. Moss-Morris R. Adjusting to chronic illness: time for a unified theory. *Br J Health Psychol* 2013; **18(4)**: 681–6.

30. Lopes AA, Bragg J, Young E, Goodkin D, Mapes D, Combe C et al. Depression as a predictor of mortality and hospitalization among hemodialysis patients in the United States and Europe. *Kidney Int* 2002; **62(1)**: 199-207.

For peer review only - http://bmjopen.bmj.com/site/about/guidelines.xhtml

FIGURE LEGEND

Figure 1: Surveys mailed and returned

For peer review only

For peer review only - http://bmjopen.bmj.com/site/about/guidelines.xhtml

TABLES

Table 1: Comparison of the characteristics of survey responders and non-responders

Characteristic	Respondents (%)	Non-respondents (%)	Total	Comparison of proportions
Stage on ESRD pathway				
Pre-RRT	183 (24.6)	560 (75.4)	743	
Dialysis <2 years	162 (25.2)	481 (74.8)	643	**X^2 = 9.96; p=0.02**
Dialysis 2+ years	293 (30.0)	684 (70.0)	977	
Transplant	402 (29.4)	965 (70.6)	1367	
Age group†				
Less than 65	503 (24.7)	1537 (75.3)	2040	**X^2 = 19.86; p<0.0001**
65 and above	524 (31.3)	1151 (68.7)	1675	
Gender				
Male	635 (28.0)	1636 (72.0)	2271	X^2 = 0.01; p=0.92
Female	405 (27.8)	1054 (72.2)	1459	
Ethnicity‡				
White	902 (34.4)	1720 (65.6)	2622	**X^2 = 76.16; p<0.0001**
Black and minority ethnic	138 (17.9)	635 (82.1)	773	

† Age unknown for 15 patients; ‡ Ethnicity unknown for 335 patients; bold text indicates statistical significance

For peer review only

For peer review only - http://bmjopen.bmj.com/site/about/guidelines.xhtml

Table 2: Respondent characteristics and proportion by sub-group with mild to moderate distress

Characteristic	Respondents (%)	Mild to moderate distress (%)	Comparison of proportions (mild to moderate distress patients)†
All respondents	1040 (100.0)	346 (33.3)	
Study site			
Site 1	187 (18.0)	72 (38.5)	
Site 2	177 (17.0)	58 (32.8)	X^2 = 3.24; p=0.356
Site 3	323 (31.1)	107 (33.1)	
Site 4	353 (33.9)	109 (30.9)	
Stage on ESRD pathway			
Pre-RRT	182 (17.5)	64 (35.2)	
Dialysis <2 years	154 (14.8)	55 (35.7)	X^2 = 4.85; p=0.183
Dialysis 2+ years	300 (28.8)	109 (36.3)	
Transplant	404 (38.8)	118 (29.2)	
Dialysis type (n=454)			
Hospital/in-centre haemodialysis	343 (75.6)	129 (37.6)	
Home haemodialysis	31 (6.8)	13 (41.9)	X^2 = 3.36; p=0.186
Peritoneal dialysis	80 (17.6)	22 (27.5)	
Age group‡			
Less than 50	174 (16.9)	78 (44.8)	
50 to 69	441 (42.9)	145 (32.9)	**X^2 = 14.33; p=0.0008**
70 and above	414 (40.2)	119 (28.7)	
Gender			
Male	633 (60.9)	191 (30.2)	**X^2 = 6.63; p=0.01**
Female	407 (39.1)	155 (38.1)	
Ethnicity			
White	902 (86.7)	283 (31.4)	**X^2 = 10.36; p=0.0013**
Black and minority ethnic	138 (13.3)	63 (45.7)	

†Bold text denotes a statistically significant difference between groups; ‡Age unknown for four respondents

For peer review only - http://bmjopen.bmj.com/site/about/guidelines.xhtml

Table 3: Perceived need for help with distress by sub-group with mild to moderate distress (n=346)

Characteristic	Median 'need' score (IQR)	Patients scoring 4 or more on need thermometer (%)	Comparison of proportions (mild to moderate distress patients)†
All respondents	3 (1 to 5)	141 (40.8)	
Study site			
Site 1	1 (0 to 4)	55 (29.4)	
Site 2	1 (0 to 3)	38 (21.5)	**X^2 = 9.49; p=0.02**
Site 3	1 (0 to 3)	74 (22.9)	
Site 4	0 (0 to 3)	71 (20.1)	
Stage on ESRD pathway			
Pre-RRT	2 (0 to 4)	22 (34.9)	
Dialysis <2 years	3 (2 to 6)	35 (66.0)	**X^2 = 27.71; p<0.0001**
Dialysis 2+ years	4 (2 to 6)	59 (55.7)	
Transplant	2 (0 to 4)	35 (29.9)	
Dialysis type (n=454)			
Hospital/in-centre haemodialysis	4 (2 to 6)	70 (56.0)	
Home haemodialysis	4 (2 to 8)	7 (53.8)	X^2 = 5.44; p=0.07
Peritoneal dialysis	3 (0 to 5)	6 (28.6)	
Age group			
Less than 50	3 (2 to 5)	35 (44.9)	
50 to 69	3 (1 to 5)	55 (39.0)	X^2 = 0.85; p=0.654
70 and above	3 (1 to 5)	48 (41.4)	
Gender			
Male	3 (1 to 5)	73 (39.2)	X^2 = 0.54; p=0.462
Female	3 (1 to 5)	67 (43.8)	
Ethnicity			
White	3 (1 to 5)	106 (37.9)	**X^2 = 7.06; p=0.008**
Black and minority ethnic	5 (2 to 7)	34 (57.6)	

†Bold text indicates a statistically significant difference between groups

For peer review only

Table 4: Number and proportion of patients reporting problems on the DT problem list

Problems by domain	Number of patients (%)†	Patients reporting at least 1 problem for the domain (%)
Any problems (n=36)	-	339 (98.0)
Practical domain (n=5)		
Transport	84 (24.3)	
Insurance/financial	76 (22.0)	
Work	53 (15.3)	167 (48.3)
Housing	30 (8.7)	
Childcare	13 (3.8)	
Family domain (n=3)		
Dealing with friend/relative	66 (19.1)	
Dealing with partner	60 (17.3)	118 (34.1)
Dealing with children	27 (7.8)	
Emotional domain (n=6)		
Worry	257 (74.3)	
Loss of interest in usual activities	186 (53.8)	
Sadness	166 (48.0)	318 (91.9)
Depression	159 (46.0)	
Nervousness	150 (43.4)	
Fears	148 (42.8)	
Spiritual domain (n=1)		
Spiritual/religious concerns	23 (6.6)	-
Physical domain (n=21)		
Fatigue	248 (71.7)	
Skin dry/itchy	225 (65.0)	
Sleep	212 (61.3)	
Memory/concentration	183 (52.9)	
Pain	178 (51.4)	
Getting around	171 (49.4)	
Tingling in hands/feet	144 (41.6)	
Breathing	133 (38.4)	
Feeling swollen	127 (36.7)	
Bathing/dressing	119 (34.4)	329 (95.1)
Appearance	116 (33.5)	
Eating	111 (32.1)	
Nose dry/congested	108 (31.2)	
Constipation	106 (30.6)	
Nausea	100 (28.9)	
Changes in urination	94 (27.2)	
Indigestion	92 (26.6)	
Sexual	88 (25.4)	
Diarrhoea	79 (22.8)	
Mouth sores	42 (12.1)	
Fevers	31 (9.0)	

†Problems within each domain ranked from most to least frequently cited by respondents

For peer review only

For peer review only - http://bmjopen.bmj.com/site/about/guidelines.xhtml

Table 5: Median number of problems reported in each DT problem list domain, by patient group

Characteristic	Practical†,‡	Family	Emotional	Physical	Total
Stage on ESRD pathway	Median (IQR)§	Median (IQR)	Median (IQR)	Median (IQR)	Median (IQR)
Pre-RRT	1 (0 to 1)	0 (0 to 1)	3 (1 to 4)	**8 (4 to 12)**	**12 (7 to 17)**
Dialysis <2 years	1 (0 to 2)	0 (0 to 1)	3 (2 to 5)	**8 (6 to 12)**	**13 (9 to 17)**
Dialysis 2+ years	1 (0 to 1)	0 (0 to 1)	3 (2 to 5)	**9 (6 to 11)**	**13 (9 to 16)**
Transplant	0 (0 to 1)	0 (0 to 1)	3 (1 to 4)	**6 (3 to 10)**	**10 (5 to 16)**
				**p=0.019**	**p=0.023**
Dialysis type (n=164)					
Hospital/in-centre haemodialysis	1 (0 to 1)	0 (0 to 1)	3 (2 to 5)	8 (5 to 11)	13 (8 to 16)
Home haemodialysis	1 (0 to 2)	0 (0 to 1)	4 (2 to 5)	10 (8 to 13)	16 (11 to 19)
Peritoneal dialysis	0 (0 to 1)	0 (0 to 1)	3 (2 to 4)	8 (6 to 11)	12 (9 to 16)
Age group[4]					
Less than 50	**1 (0 to 2)**	**0 (0 to 1)**	**3 (2 to 5)**	7 (4 to 11)	**13 (8 to 17)**
50 to 69	**1 (0 to 2)**	**0 (0 to 1)**	**3 (2 to 5)**	8 (5 to 11)	**12 (8 to 18)**
70 and above	**0 (0 to 1)**	**0 (0 to 0)**	**3 (1 to 4)**	7 (4 to 10)	**11 (7 to 15)**
	**p<0.0001**	**p=0.001**	**p=0.038**		**p=0.033**
Gender					
Male	1 (0 to 1)	0 (0 to 1)	3 (2 to 4)	**7 (4 to 10)**	12 (7 to 16)
Female	0 (0 to 1)	0 (0 to 1)	3 (2 to 5)	**8 (5 to 12)**	12 (8 to 17)
				**p=0.043**	
Ethnicity					
White	0 (0 to 1)	0 (0 to 1)	3 (2 to 4)	8 (5 to 11)	12 (7 to 16)
Black and minority ethnic	0 (0 to 2)	0 (0 to 1)	4 (1 to 5)	7 (4 to 10)	11 (7 to 17)
All respondents (n=346)	0 (0 to 1)	0 (0 to 1)	3 (2 to 5)	8 (5 to 11)	12 (7 to 16)

†Bold text denotes a statistically significant difference between groups (Mann Whitney test for characteristics with two sub-groups; Kruskall Wallis test for characteristics with three or more sub-groups); ‡Spiritual domain excluded from analysis; §IQR = inter-quartile range

For peer review only - http://bmjopen.bmj.com/site/about/guidelines.xhtml

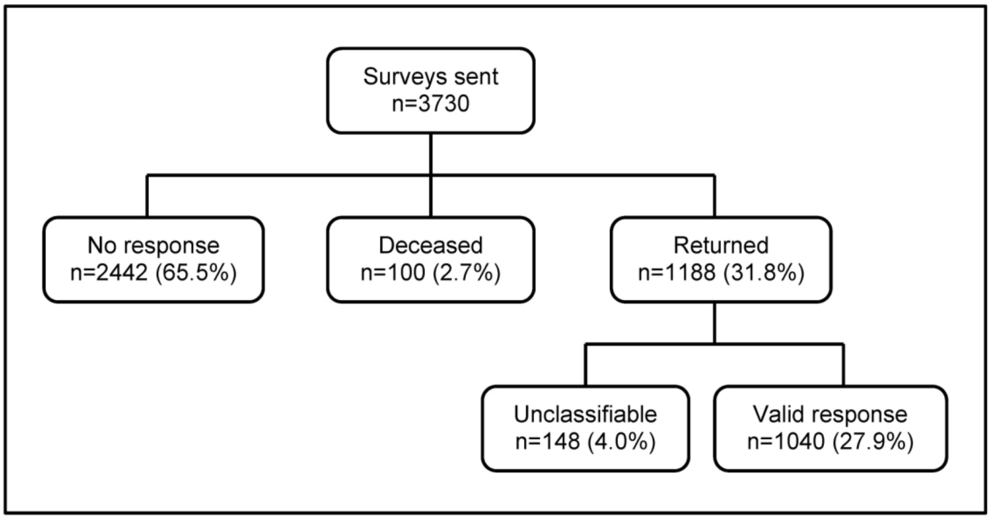

Figure 1: Surveys mailed and returned

150x78mm (300 x 300 DPI)

STROBE 2007 (v4) Statement—Checklist of items that should be included in reports of *cross-sectional studies*

Section/Topic	Item #	Recommendation	Reported on page #
**Title and abstract**	1	(*a*) Indicate the study's design with a commonly used term in the title or the abstract	1
		(*b*) Provide in the abstract an informative and balanced summary of what was done and what was found	2
**Introduction**			
Background/rationale	2	Explain the scientific background and rationale for the investigation being reported	4
Objectives	3	State specific objectives, including any prespecified hypotheses	4
**Methods**			
Study design	4	Present key elements of study design early in the paper	5-6
Setting	5	Describe the setting, locations, and relevant dates, including periods of recruitment, exposure, follow-up, and data collection	4-5
Participants	6	(*a*) Give the eligibility criteria, and the sources and methods of selection of participants	5
Variables	7	Clearly define all outcomes, exposures, predictors, potential confounders, and effect modifiers. Give diagnostic criteria, if applicable	5-6
Data sources/ measurement	8*	For each variable of interest, give sources of data and details of methods of assessment (measurement). Describe comparability of assessment methods if there is more than one group	5-6
Bias	9	Describe any efforts to address potential sources of bias	*5*
Study size	10	Explain how the study size was arrived at	6
Quantitative variables	11	Explain how quantitative variables were handled in the analyses. If applicable, describe which groupings were chosen and why	5-6
Statistical methods	12	(*a*) Describe all statistical methods, including those used to control for confounding	5-6
		(*b*) Describe any methods used to examine subgroups and interactions	5-6
		(*c*) Explain how missing data were addressed	6
		(*d*) If applicable, describe analytical methods taking account of sampling strategy	N/A
		(*e*) Describe any sensitivity analyses	N/A
**Results**			

For peer review only

Participants	13*	(a) Report numbers of individuals at each stage of study—eg numbers potentially eligible, examined for eligibility, confirmed eligible, included in the study, completing follow-up, and analysed	6; Fig 1
		(b) Give reasons for non-participation at each stage	N/A
		(c) Consider use of a flow diagram	Fig 1
Descriptive data	14*	(a) Give characteristics of study participants (eg demographic, clinical, social) and information on exposures and potential confounders	6
		(b) Indicate number of participants with missing data for each variable of interest	N/A
Outcome data	15*	Report numbers of outcome events or summary measures	6-7
Main results	16	(*a*) Give unadjusted estimates and, if applicable, confounder-adjusted estimates and their precision (eg, 95% confidence interval). Make clear which confounders were adjusted for and why they were included	6-7
		(*b*) Report category boundaries when continuous variables were categorized	6-7
		(*c*) If relevant, consider translating estimates of relative risk into absolute risk for a meaningful time period	N/A
Other analyses	17	Report other analyses done—eg analyses of subgroups and interactions, and sensitivity analyses	6-7
**Discussion**			
Key results	18	Summarise key results with reference to study objectives	8
Limitations	19	Discuss limitations of the study, taking into account sources of potential bias or imprecision. Discuss both direction and magnitude of any potential bias	9
Interpretation	20	Give a cautious overall interpretation of results considering objectives, limitations, multiplicity of analyses, results from similar studies, and other relevant evidence	8
Generalisability	21	Discuss the generalisability (external validity) of the study results	9-10
**Other information**			
Funding	22	Give the source of funding and the role of the funders for the present study and, if applicable, for the original study on which the present article is based	11

*Give information separately for cases and controls in case-control studies and, if applicable, for exposed and unexposed groups in cohort and cross-sectional studies.

Note: An Explanation and Elaboration article discusses each checklist item and gives methodological background and published examples of transparent reporting. The STROBE checklist is best used in conjunction with this article (freely available on the Web sites of PLoS Medicine at http://www.plosmedicine.org/, Annals of Internal Medicine at http://www.annals.org/, and Epidemiology at http://www.epidem.com/). Information on the STROBE Initiative is available at www.strobe-statement.org.

For peer review only

BMJ Open

The prevalence of mild to moderate distress in patients with end stage renal disease: results from a patient survey using the emotion thermometers in four hospital Trusts in the West Midlands, UK

Journal:	*BMJ Open*
Manuscript ID	bmjopen-2018-027982.R2
Article Type:	Research
Date Submitted by the Author:	10-Apr-2019
Complete List of Authors:	Damery, Sarah; University of Birmingham, Institute of Applied Health Research<br>Brown, Celia ; University of Warwick<br>Sein, Kim; University of Birmingham, Institute of Applied Health Research<br>Nicholas, Johann; Shrewsbury and Telford Hospital NHS Trust, Renal Unit<br>Baharani, Jyoti; Heart of England NHS Foundation Trust, Renal Medicine<br>Combes, Gill; University of Birmingham, Institute of Applied Health Research
**Primary Subject Heading**:	Renal medicine
Secondary Subject Heading:	Epidemiology, Communication, Health services research
Keywords:	survey, Chronic renal failure < NEPHROLOGY, distress, End stage renal failure < NEPHROLOGY, emotion thermometers

SCHOLARONE™
Manuscripts

TITLE

The prevalence of mild to moderate distress in patients with end stage renal disease: results from a patient survey using the emotion thermometers in four hospital Trusts in the West Midlands, UK.

AUTHORS

Sarah Damery,* Research Fellow, Institute of Applied Health Research, University of Birmingham, Edgbaston, West Midlands, B15 2TT, United Kingdom. Email: s.l.damery@bham.ac.uk; Tel: +44 (0)121 414 3343 [Corresponding author]

Celia Brown,[§] Associate Professor, Warwick Medical School, University of Warwick, Coventry, CV4 7AL, United Kingdom. Email: celia.taylor@warwick.ac.uk

Kim Sein, Research Fellow, Institute of Applied Health Research, University of Birmingham, Edgbaston, West Midlands, B15 2TT, United Kingdom. Email: k.sein@bham.ac.uk

Johann Nicholas, Consultant Nephrologist, Renal Unit, Shrewsbury and Telford NHS Trust, Mytton Oak Road, Shrewsbury, SY3 8XQ, United Kingdom. Email: johann.nicholas@nhs.net

Jyoti Baharani, Consultant Nephrologist, Renal Unit, Birmingham Heartlands Hospital, Heart of England NHS Foundation Trust, Bordesley Green East, Birmingham, B9 5SS, United Kingdom. Email: jyoti.baharani@heartofengland.nhs.uk

Gill Combes, Senior Research Fellow, Institute of Applied Health Research, University of Birmingham, Edgbaston, West Midlands, B15 2TT, United Kingdom. Email: g.combes@bham.ac.uk

[§] Celia Taylor at the time the research was undertaken

For peer review only

For peer review only - http://bmjopen.bmj.com/site/about/guidelines.xhtml

ABSTRACT

Objectives

To assess the prevalence of mild to moderate distress in patients with end stage renal disease and determine the association between distress and patient characteristics.

Design

Cross-sectional survey using the emotion thermometers and distress thermometer problem list.

Setting

Renal units in four hospital Trusts in the West Midlands, UK.

Participants

Adult patients with stage 5 chronic kidney disease who were: i) pre-renal replacement therapy; ii) on dialysis for less than 2 years; iii) on dialysis for 2 or more years, or iv) with a functioning transplant.

Outcomes

The prevalence of mild to moderate distress, and the incidence of distress thermometer problems and patient support needs.

Results

In total, 1040/3730 surveys were returned (27.9%). One third of survey respondents met the criteria for mild to moderate distress (n=346; 33.3%). Prevalence was highest in patients on dialysis for two or more years (n=109/300; 36.3%) and lowest in transplant patients (n=118/404; 29.2%). Prevalence was significantly higher in younger vs. older patients (X^2=14.33; p=0.0008), in females vs. males (X^2=6.63; p=0.01) and in black and minority ethnic patients vs. patients of white ethnicity (X^2=10.36; p=0.013). Over forty per cent of patients (n=141) reported needing support. More than 95% of patients reported physical problems and 91.9% reported at least one emotional problem.

Conclusions

Mild to moderate distress is common in patients with end stage renal disease, and there may be substantial unmet support needs. Regular screening could help identify patients whose distress may otherwise remain undetected. Further research into differences in distress prevalence over time and at specific transitional points across the renal disease pathway is needed, as is work to determine how best to support patients requiring help.

KEY WORDS

Chronic Kidney Disease, Distress, End Stage Renal Disease, emotion thermometers, survey

For peer review only

STRENGTHS AND LIMITATIONS OF THIS STUDY

- This study is the first to explore the prevalence of mild to moderate distress in a large cohort of patients with end stage renal disease (ESRD)
- Findings suggest that distress affects around a third of patients with ESRD at any one time, and that there are substantial unmet support needs in this population
- The inclusion of multiple study sites is likely to have minimised any bias arising from variations in the organisation and delivery of renal services
- The survey response rate was low, and younger patients, those from black and minority ethnic (BME) groups and patients who had been more recently diagnosed were under-represented in responses.

For peer review only

For peer review only - http://bmjopen.bmj.com/site/about/guidelines.xhtml

BACKGROUND

In 2016, there were 51,672 patients with end stage renal disease (ESRD) receiving renal replacement therapy (RRT) in England.[1] Treatment is life-sustaining but not curative, and patients must constantly adjust to frequent changes to health status and likelihood of survival. Consequently, patients can experience many emotional and psychological stressors, including acceptance of diagnosis, disease progression, choosing treatment options, coping with dialysis, and associated impacts on employment, relationships and lifestyle.[2,3] Evidence suggests that patients with ESRD experience rates of depression and anxiety markedly higher than the general population.[4,5] Establishing prevalence is challenging, partly because many uraemic symptoms can be misinterpreted as symptoms of depression,[6] and partly due to the variation in prevalence estimates obtained using different diagnostic tools and modes of assessment (e.g. self-reported vs. interview-based scales).[7] Consequently, depression and anxiety prevalence estimates reported in the ESRD literature range from around 6% to 71%,[8] but are generally considered to be between 20 to 30% for dialysis patients[9] and around 25% for transplant patients.[10] This compares to a point prevalence of depression of between 2 and 9% and lifetime depression risk of around 7% in the general population.[11]

Untreated anxiety and depression in patients with ESRD are associated with decreased health-related quality of life and higher symptom burden.[12] These factors may raise the risk of poor outcomes, increased healthcare use, and sub-optimal adherence with diet and medication regimes.[13-15] There is also evidence that depression status is associated with an elevated risk of all-cause mortality in renal patients, with meta-analysis suggesting an excess mortality risk attributable to depression higher than that observed in other chronic diseases such as cancer and diabetes.[7] Provision of emotional and psychological support is central to the recommended management of renal disease, and both the United Kingdom Department of Health and NICE (National Institute for Health and Care Excellence) mandate provision of such support within national renal guidelines.[16,17]

Whilst supportive services are relatively advanced for those with higher level needs requiring psychiatric or psychological intervention, there is a lack of robust data on the prevalence of lower-level support needs – defined as difficulties adjusting to, and coping effectively with, renal failure, diagnosis, physical symptoms and treatment. It may also be useful to broaden the focus beyond narrowly-defined anxiety and depression to consider the determinants and consequences of more general emotional and psychological difficulty encompassed by the term ‘distress’. Whilst linear progression from lower-level to severe difficulty is not inevitable, timely identification of patient distress may facilitate effective management. This study aimed to assess the prevalence of mild to moderate distress in a cohort of patients with ESRD and determine the association between distress and sociodemographic and clinical characteristics. It forms part of a larger mixed methods study that aims to understand how the recognition and management of patients’ emotional and psychological support needs can be integrated into the ESRD pathway.[18]

METHODS

The study used a cross-sectional survey, distributed by post to all eligible patients with ESRD managed at four National Health Service (NHS) hospital Trusts in the West Midlands, UK. Trusts were chosen to maximise diversity in patient demographics, catchment size, urban-rural mix and the organisation of psychological support services: site 1 (small) and site 2 (large) both serve urban, inner city catchments with

For peer review only - http://bmjopen.bmj.com/site/about/guidelines.xhtml

substantial black and minority ethnic (BME) populations. Site 3 (medium) and site 4 (large) both serve predominantly white populations in urban areas with surrounding rural districts. Sites 2 and 4 have access to a renal psychologist for the provision of psychological support services, whereas sites 1 and 3 do not.

Participants and recruitment

Eligible patients were aged 18+ years, diagnosed with chronic kidney disease (CKD) stage 5, and grouped according to their stage on the ESRD pathway: i) pre-RRT, ii) on peritoneal or haemodialysis for less than two years, iii) on dialysis for two or more years, or iv) with a functioning transplant. Although differentiating according to dialysis vintage is not a recognised clinical distinction, it was hypothesised by the clinicians involved in designing this study that there may be differences in distress prevalence between patients initiating dialysis more recently vs. those undergoing dialysis for longer. Patients using psychiatric services since CKD5 diagnosis were excluded. Renal unit staff at each Trust identified eligible patients from hospital records, and survey packs were prepared on Trust premises by the University research team. Eligible patients received a letter of invitation from the renal unit's lead consultant, an information sheet and a survey, to be returned directly to the researchers. Return envelopes were marked with a unique identifier for recording returns, and non-responders received one reminder after six weeks. Mailings were carried out between January 2016 and May 2017.

Survey

The survey measured distress with the Emotion Thermometers (ET),[19,20] which use a visual analogue scale covering five domains: distress, anxiety, depression, anger and perceived need for help. Patients score each domain on an 11-point Likert scale to rate their levels of emotional upset during the preceding week, where '0' corresponds to none and '10' denotes extreme problems. Although not validated specifically for use with renal patients, the ET has been widely used in studies of patients with cancer and other chronic conditions where it has been found to be sensitive in identifying emotional difficulty and broadly defined distress.[19] It incorporates the distress thermometer (DT), which has been validated in the UK population with chronic kidney disease.[21] The survey also included the Distress Thermometer Problem List[22] which lists 36 problems across five domains: practical (n=5), family (n=3), emotional (n=6), spiritual (n=1) and physical (n=21). Patients indicated which (if any) of the 36 problems they had experienced in the previous week. Closed questions covered sociodemographic characteristics (age, sex, ethnicity) and treatment modality (where relevant).

Data analysis

Thresholds for distress using the ET have been validated,[19,20] with a score of 4 to 5 denoting mild distress, and 6 to 7 denoting moderate distress. Patients were defined as having mild to moderate distress if they: a) scored between 4 to 7 on the DT (regardless of scores in the other ET domains), or b) scored 0 to 3 on the DT and 4 to 7 on one or more of the anxiety, depression and anger thermometers, with no thermometer exceeding 7. Analysis was descriptive, focusing on associations between distress and respondents' stage on the ESRD pathway and sociodemographic characteristics. Anonymised data were obtained from hospital information systems to allow a comparison of the characteristics of respondents and non-respondents on the basis of age group, sex, ethnic group and ESRD pathway stage. Comparisons were undertaken using chi-squared analysis. Any surveys in which the emotion thermometers were left blank by a respondent were

For peer review only - http://bmjopen.bmj.com/site/about/guidelines.xhtml

excluded from analysis. The prevalence of total and individual problems from the DT problem list were analysed descriptively, and medians and inter-quartile ranges were calculated to compare numbers of problems cited within each domain. Data were analysed using SPSS (version 21.0, Armonk, NY: IBM Corp).

Sample size

The primary outcome was the difference in the prevalence of mild to moderate distress across patients at different ESRD stages. We anticipated an average prevalence of 25% across all patients,[9] with patients in the stages with the highest and lowest prevalence at +/- 5 percentage points from this average (i.e. 20% for the stage with the lowest prevalence and 30% for the stage with the highest prevalence). This equated to a small effect size (w) of approximately 0.1. To detect this difference with 80% power and 5% significance, a total of 1090 responses were required (assuming approximately equal numbers of patients in each ESRD stage).

Ethical approval

Ethics approval was obtained from the Coventry and Warwickshire Research Ethics Committee in October 2015 [Ref 15/WM/0288]. The study was also approved by the Research Governance office of each of the participating hospital Trusts.

Patient and public involvement

The study design and research questions were developed with input from a study advisory group that included patient representatives, and the patient and public involvement (PPI) group attached to the chronic diseases theme of the CLAHRC West Midlands. These groups were also involved in selecting appropriate outcome measures that optimised data quality whilst minimising participant burden. Patients were not involved in recruitment to the study or its conduct. Study participants will be sent a plain English summary of final study results if they indicated during the informed consent process that they would like to receive this.

RESULTS

A total of 3730 surveys were sent across the four study sites. One hundred patients died between the initial and reminder mailings (2.7%) and 2442 recipients (65.5%) did not respond. Of the 1188 responses received (31.8%), 148 were removed due to non-completion of the emotion thermometers (4.0%), giving a total of 1040 valid responses (27.9%) (Figure 1). Rates of valid responses ranged from 23.0% in site 1 to 30.4% in site 4. Younger patients (<65 years old) were significantly less likely to respond than those aged 65 and over, as were those from BME groups compared to those in the white ethnic group. Patients yet to begin RRT and those on dialysis for less than 2 years were significantly less likely to respond than patients with a transplant or who had been undergoing dialysis for 2 or more years. There was no significant difference between responders and non-responders on the basis of sex (Table 1).

Characteristics of respondents

The majority of respondents were male (n=633; 60.9%) and in the white ethnic group (n=902; 86.7%) (Table 2). Patients aged between 51 and 69 years old constituted the largest age group (n=441; 42.9%), with those aged under 50 comprising 16.9% of the total (n=174). Nearly two fifths of respondents had received a transplant (n=404; 38.8%) and 28.8% had been on dialysis for 2 or more years (n=300). Of the 454 patients

For peer review only - http://bmjopen.bmj.com/site/about/guidelines.xhtml

undergoing regular dialysis, the most common modality was hospital/in-centre haemodialysis (n=343; 75.6%).

Prevalence of distress

A total of 561 respondents (53.9%) were categorised as having none to low distress, and a further 133 patients (12.3%) met the criteria for severe distress (i.e. scoring greater than 7 on one or more of the emotion thermometers). A total of 346 respondents (33.3%) met the criteria for mild to moderate distress (hereafter termed 'distress'). Distress prevalence by hospital site was lowest in site 4 (30.9%) and highest in site 1 (38.5%), although the difference between study sites was not statistically significant (X^2 = 3.24; p=0.356). Distress was identified in 208 patients (60.1%) on the basis of their DT score alone, with the remaining 138 (39.9%) identified from their scores on the anxiety, depression or anger thermometers. Distress prevalence was highest in patients who had been on dialysis for 2 or more years (n=109/300; 36.3%) and lowest in transplant patients (n=118/404; 29.2%). For dialysis modality, distress was most prevalent for home haemodialysis patients (n=13/31; 41.9%) and lowest in the peritoneal dialysis group (n=22/80; 27.5%), although numbers were small. There was no significant difference in rates of distress on the basis of ESRD stage or dialysis type when groups were compared. In contrast, all sociodemographic characteristics showed significant differences in distress prevalence between groups. Patients in the youngest age group were significantly more likely to report distress than those in the older age groups (n=78/174; 44.8%; X^2 = 14.33; p=0.0008). The prevalence of distress was significantly higher for females than males (38.1% vs. 30.2%; X^2 = 6.63; p=0.01) and for BME patients compared to white patients (45.7% vs. 31.4%; X^2 = 10.36; p=0.0013).

Perceived need for help

Scores on the 'need' thermometer were assessed as an outcome measure indicating patients' perceived need for help. Scores of four and above were considered to indicate unmet support needs[19] (Table 3). Patients at study sites 1 and 3 had significantly higher perceived support needs than those in sites 2 and 4 (29.4% and 22.9% vs. 21.5% and 20.1%; X^2 = 9.49; p=0.02). There were also significant differences by sub-group according to ESRD stage: 66.0% of patients on dialysis for less than two years (n=35) required support, as did 55.7% of patients on dialysis for two or more years (n=59). Perceived support needs in the pre-RRT and transplant groups were significantly lower, at 34.9% and 29.9% respectively (X^2 = 27.71; p<0.0001). Support needs were also significantly higher in BME compared to white patients, at 57.6% vs. 37.9% (X^2 = 7.06; p=0.008).

Distress thermometer problem list

All but six patients reported experiencing at least one problem on the DT problem list in the preceding week (98.0%) (Table 4). The most frequently reported problems were all in the emotional and physical domains, with 91.9% of patients reporting at least one emotional problem in the previous week, and 95.1% reporting at least one physical problem. 'Worry' was the commonest problem across all domains (n=247; 74.3%). 'Fatigue' (71.7%), 'dry/itchy skin' (65.0%) and 'sleep' (61.3%) were the most common physical problems. 'Spiritual problems' (6.6%) and 'problems with childcare' (family domain: 3.8%) were reported least frequently. The median number of total problems reported was 12/36 (IQR: 7 to 16) (Table 5).

For peer review only - http://bmjopen.bmj.com/site/about/guidelines.xhtml

Sub-group analysis shows that transplant patients reported significantly fewer physical problems and total problems than pre-RRT or dialysis patients (median physical problems 6/21, IQR 3 to 10, p=0.019; median total problems 10/36, IQR 5 to 16, p=0.023). Patients aged 70 and older reported significantly fewer problems on the practical, family and emotional domains than younger patients. They also reported significantly fewer total problems. Females reported significantly more physical problems than males (median 8/21, IQR 5 to 12 vs. 7/21, IQR 5 to 11; p=0.043).

DISCUSSION

Detecting distress is important for the optimal care of patients with ESRD, and evidence suggests that reducing emotional and psychological difficulties may enhance wellbeing and improve patients' ability to engage with complex and demanding treatments. Our study has shown that as many as one in three patients with ESRD may have mild to moderate distress, and rates of reporting emotional and physical problems were high. This was evident across the ESRD pathway, with around 35% of pre-RRT and dialysis patients and 29% of transplant patients meeting the criteria for mild to moderate distress. The finding that the prevalence of distress was almost as high in transplant patients as those undergoing dialysis suggests that although they may experience fewer physical problems, the need for ongoing psychological adjustment does not end at the point of transplantation.[10] Mild to moderate distress was most prevalent in the group of patients who had been on dialysis for 2 or more years (36.3%), which may reflect a lack of adjustment and coping in this group with the ongoing challenges of undergoing regular dialysis treatment over an extended period, potentially declining health, and the limitations that dialysis treatment places on family, relationships and lifestyle. There was also variation by dialysis type, with patients receiving hospital/in-centre haemodialysis reporting distress rates of 37.6%, compared to 27.5% for patients undergoing peritoneal dialysis. Numbers were too small to detect statistically significant differences, but this distinction has been found elsewhere,[23] and would benefit from further study.

Distress prevalence was strongly associated with sociodemographic characteristics and was significantly higher in younger vs. older patients, in BME vs. white patients, and in females compared to males. These trends have also been found in studies of anxiety and depression in renal patients.[24,25] There are numerous psychological theories of health and illness which may have a role in explaining patients' variable responses to ESRD diagnosis and treatment.[26-28] These theories emphasise that experience of distress is likely to be determined by an individual's personal degree of resilience and individual coping resources rather than being associated with specific clinical characteristics.

Comparable national figures for the pre-RRT group are not available, but if the prevalence of mild to moderate distress found in this study was standardised to the current population in England with a functioning transplant or undergoing dialysis, it would equate to 18,970 patients with ESRD experiencing difficulties, of whom 7,835 may want help.[1] It has been argued that the primary goal of supportive services should be to distinguish between transient and ongoing difficulties and focus on optimising patients' ability to cope with their condition.[29] In this context, it is likely that the most feasible supportive interventions are those that can be incorporated into routine clinical practice and standard protocols for ESRD care. The ET could be used regularly by clinicians during patient consultations or by renal nurses to screen for distress. Screening

For peer review only - http://bmjopen.bmj.com/site/about/guidelines.xhtml

in itself will not necessarily lead to better patient outcomes, but it may help to identify patients whose distress would otherwise remain undetected, and in doing so, could enable the targeted provision of support services.

Experiencing distress may be considered a normal response to chronic disease diagnosis, and not all patients with mild to moderate distress will want to receive support. The likelihood that patients with ESRD will experience symptoms of distress was predominantly associated with age, sex and ethnicity. Consequently, screening for distress would seem particularly important for younger patients, who may experience significantly greater disruption to their family and working life than older patients, females, and for patients in BME groups, who reported uniformly high rates of mild to moderate distress and high support needs. It may also be useful to target screening for distress towards those who undergo regular hospital or in-centre haemodialysis. Providing appropriate information to patients early on in the ESRD pathway (pre-RRT) about the distress they may experience could also help to manage patients' expectations and allow timely identification of emotional and psychological difficulties. There was also evidence of a centre effect in the proportion of patients reporting a perceived need for support at participating study sites. Patients in sites 1 and 3, where there is no access to a renal psychologist reported significantly higher rates of support need than those in sites 2 and 4 where renal psychologist support is available. This suggests that the availability of in-house psychological support services may play an important role in helping patients with ESRD to manage distress.

Limitations

The survey response rate was low, and younger patients, those from BME groups and patients who had been more recently diagnosed were under-represented in responses. The study was marginally underpowered, with 1040 valid responses received, rather than the 1090 required by our *a priori* sample size estimation. We are also unable to say whether distress is more or less common in survey respondents compared to non-respondents, nor could we perform detailed sub-group analyses for patients within each ESRD pathway stage. This meant that participants in each ESRD pathway stage were treated as a homogeneous cohort when in reality there may have been differences between them that may have impacted on their experience of distress such as issues with medication or ESRD-related complications like fistula failure, infection or transplant failure. However, in-depth qualitative research was undertaken with renal patients to explore the detailed determinants and consequences of their distress in a linked component of this study (findings to be reported elsewhere).

Nevertheless, our findings with regard to the patient groups most likely to experience distress were similar to existing evidence on anxiety and depression in renal disease. Because younger, BME patients were under-represented in our sample, the finding that patients in these groups reported significantly higher rates of mild to moderate distress than older, white patients, suggests that we may have under-estimated rather than over-estimated overall distress prevalence. The inclusion of multiple study sites is likely to have minimised any bias that may arise from variations in the organisation and delivery of renal services. Patients referred to psychiatric services (as noted in their hospital record) were excluded, but we cannot know whether our sample included patients who had independently sought counselling or support via their general practitioner. The study was also cross-sectional rather than longitudinal, and consequently allows limited understanding of the relationship between time since ESRD diagnosis and ability to cope with the resulting stressors. Some

For peer review only - http://bmjopen.bmj.com/site/about/guidelines.xhtml

studies with renal patients have found no correlation between depressive symptoms and time since treatment initiation,[9] whereas others have found a tendency for depression status to worsen.[30] Future work using a cohort study design would aid understanding of the ways that individuals adapt to ESRD diagnosis and its ongoing management over time.

CONCLUSION

This study is, to our knowledge, the first to explore the prevalence of mild to moderate distress in a large cohort of patients with ESRD. Our findings show that mild to moderate distress is common – even after transplantation – and there may be substantial unmet patient support needs. Further research into possible variations in distress prevalence over time and at different transitional points across the ESRD pathway is needed, as is work to determine how best to support patients requiring help.

For peer review only

For peer review only - http://bmjopen.bmj.com/site/about/guidelines.xhtml

DECLARATIONS

Funding

This research was funded by the United Kingdom National Institute for Health Research (NIHR) Collaboration for Leadership in Applied Health Research and Care West Midlands (CLAHRCWM). The views expressed in this manuscript are those of the authors and not necessarily those of the National Health Service, the NIHR or the Department of Health and Social Care.The funder had no role in study design, the collection, analysis and interpretation of data, or in writing the manuscript.

Competing interests

All authors declare that they have no competing interests.

Author contributions

SD managed the survey, undertook all statistical analysis and drafted the first version of the manuscript. CB contributed to research design and helped to develop the methodological/analytical approach. KS contributed to data analysis, interpretation and refinement of study methodology. JN and JB both participated in research design and in the development and refinement of study methodology. GC conceived the study, was chief investigator, and contributed to all aspects of research design and methodology. All authors have seen and approved the final version of the manuscript.

Acknowledgements

We would like to thank the renal patient advisory group and the patient and public involvement group of the chronic disease theme of CLAHRCWM for their contribution to study design and choice of data collection tools.

Exclusive licence

I, the Submitting Author has the right to grant and does grant on behalf of all authors of the Work (as defined in the below author licence), an exclusive licence and/or a non-exclusive licence for contributions from authors who are: i) UK Crown employees; ii) where BMJ has agreed a CC-BY licence shall apply, and/or iii) in accordance with the terms applicable for US Federal Government officers or employees acting as part of their official duties; on a worldwide, perpetual, irrevocable, royalty-free basis to BMJ Publishing Group Ltd ("BMJ") its licensees and where the relevant Journal is co-owned by BMJ to the co-owners of the Journal, to publish the Work in BMJ Open and any other BMJ products and to exploit all rights, as set out in our licence.

For peer review only - http://bmjopen.bmj.com/site/about/guidelines.xhtml

Data sharing

No additional data are available as research participants did not consent for their data to be shared outside of the study for which it was collected.

For peer review only

For peer review only - http://bmjopen.bmj.com/site/about/guidelines.xhtml

REFERENCES

1. Byrne C, Caskey F, Castledine C, Dawnay A, Ford D, Fraser S et al. 19th Annual Report of the Renal Association. *NEPHRON* 2017; **137** Suppl 1. UK Renal Registry: Bristol, UK.

2. Gregory DM, Way CY, Hutchinson TA, Barrett BJ, Parfrey PS. Patients' perceptions of their experiences with ESRD and hemodialysis treatment. *Qual Health Res* 1998; **8(6)**: 764-83.

3. Kimmel PL, Peterson RA. Depression in end-stage renal disease patients treated with hemodialysis: tools, correlates, outcomes, and needs. *Semin Dial* 2005; **18(2)**: 91-7.

4. Zalai D, Szeifert L, Novak M. Psychological distress and depression in patients with chronic kidney disease. *Semin Dial* 2012; **25(4)**: 428-38.

5. Hedayati SS, Yalamanchili V, Finkelstein FO. A practical approach to the treatment of depression in patients with chronic kidney disease and end-stage renal disease. *Kidney Int* 2012; **81(3)** :247-55.

6. McKercher C, Sanderson K, Jose MD. Psychosocial factors in people with chronic kidney disease prior to renal replacement therapy. *Nephrology* 2013; **18(9)**: 585-91.

7. Palmer S, Vecchio M, Craig JC, Tonelli M, Johnson DW, Nicolucci A et al. Prevalence of depression in chronic kidney disease: systematic review and meta-analysis of observational studies. *Kidney Int* 2013; **84(1)**: 179-91.

8. Chilcot J, Wellsted D, Da Silva-Gane M, Farrington K. Depression on dialysis. *Nephron Clin Pract* 2008; **108(4)**: 256-64.

9. Cukor D. Use of CBT to treat depression among patients on hemodialysis. *Psychiatr Serv* 2007; **58(5)**: 711-12.

10. Chilcot J, Spencer BW, Maple H, Mamode N. Depression and kidney transplantation. *Transplantation* 2014; **97(7)**: 717-21.

For peer review only

For peer review only - http://bmjopen.bmj.com/site/about/guidelines.xhtml

11. Cohen SD, Norris L, Acquaviva K, Peterson RA, Kimmel PL. Screening, diagnosis and treatment of depression in patients with end stage renal disease. *Clin J Am Soc Nephrol* 2007; **2(6)**: 1332-42.

12. Soni RK, Weisbord SD, Unruh ML. Health-related quality of life outcomes in chronic kidney disease. *Curr Opin Nephrol Hypertens* 2010; **19(2)**: 153-9.

13. Cukor D, Rosenthal DS, Jindal RM, Brown CD, Kimmel PL. Depression is an important contributor to low medication adherence in hemodialyzed patients and transplant recipients. *Kidney Int* 2009; **75(11)**: 1223-9.

14. Clark S, Farrington K, Chilcot J. Nonadherence in dialysis patients: prevalence, measurement, outcome, and psychological determinants. *Semin Dial* 2014; **27(1)**: 42–9.

15. Weisbord SD, Fried LF, Mor MK, Resnick AL, Unruh ML, Palevsky PM et al. Renal provider recognition of symptoms in patients on maintenance hemodialysis. *Clin J Am Soc Nephrol* 2007; **2(5)**: 960-7.

16. Department of Health. The National Service Framework for Renal Services. Part two: chronic kidney disease, acute renal failure and end of life care. London: Department of Health, 2005.

17. National Institute for Health and Care Excellence. GS5 Chronic Kidney Disease quality standard. London: NICE, 2011.

18. Taylor F, Taylor C, Baharani J, Nicholas J, Combes G. Integrating emotional and psychological support into the end-stage renal disease pathway: a protocol for mixed methods research to identify patients' lower-level support needs and how these can most effectively be addressed. *BMC Nephrol* 2016; **17**: 111.

19. Mitchell AJ, Baker-Glenn EA, Granger L, Symonds P. Can the Distress Thermometer be improved by additional mood domains? Part I. Initial validation of the Emotion Thermometers tool. *Psychooncology* 2010; **19(2)**: 125–33.

20. Mitchell AJ, Baker-Glenn EA, Granger L, Symonds P. Can the Distress Thermometer be improved by additional mood domains? Part II. What is the optimal combination of Emotion Thermometers? *Psychooncology* 2010; **19(2)**: 134–40.

For peer review only - http://bmjopen.bmj.com/site/about/guidelines.xhtml

For peer review only

21. Alston H, Vickerstaff V, Low J, Beaty C, Da Silva Gane M, Burns A. Validation of the distress thermomets in a UK renal population. *Nephrol Dial Transplant* 2015; **30(Supplement 3)**: iii612.

22. Holland JC, Bultz BD. The NCCN guideline for distress management: a case for making distress the sixth vital sign. *J Natl Compr Canc* Netw 2007; **5(1)**: 3-7.

23. Kalender B, Ozdemir C, Dervisoglu E, Ozdemir O. Quality of life in chronic kidney disease: effects of treatment modality, depression, malnutrition and inflammation. *Int J Clin Pract* 2007; **61(4)**: 569-76.

24. Drayer RA, Piraino B, Reynolds CF III, Houck PR, Mazumder S, Bernardini J et al. Characteristics of depression in hemodialysis patients: symptoms, quality of life and mortality risk. *Gen Hosp Psychiatry* 2006; **28(4)**: 306–12.

25. Harwood L, Wilson B, Locking-Cusolito H, Sontrop J, Spittal J. Stressors and coping in individuals with chronic kidney disease. *Nephrol Nurs J* 2009; **36(3)**: 265-76.

26. Cvengros JA, Christensen AJ, Lawton WJ. Health locus of control and depression in chronic kidney disease: a dynamic perspective. *J Health Psychol* 2005; **10(5)**: 677-86.

27. Birmele B, Le Gall A, Sautenet B, Aguerre C, Camus V. Clinical, sociodemographic, and psychological correlates of health-related quality of life in chronic hemodialysis patients. *Psychosomatics* 2012; **53(1)**: 30-7.

28. Chilcot J, Wellsted D, Davenport A, Farrington K. Illness representations and concurrent depression symptoms in haemodialysis patients. *J Health Psychol* 2011; **16(7)**: 1127-37.

29. Moss-Morris R. Adjusting to chronic illness: time for a unified theory. *Br J Health Psychol* 2013; **18(4)**: 681–6.

30. Lopes AA, Bragg J, Young E, Goodkin D, Mapes D, Combe C et al. Depression as a predictor of mortality and hospitalization among hemodialysis patients in the United States and Europe. *Kidney Int* 2002; **62(1)**: 199-207.

For peer review only - http://bmjopen.bmj.com/site/about/guidelines.xhtml

For peer review only

FIGURE LEGEND

Figure 1: Surveys mailed and returned

For peer review only

For peer review only - http://bmjopen.bmj.com/site/about/guidelines.xhtml

TABLES

Table 1: Comparison of the characteristics of survey responders and non-responders

Characteristic	Respondents (%)	Non-respondents (%)	Total	Comparison of proportions
Stage on ESRD pathway				
Pre-RRT	183 (24.6)	560 (75.4)	743	
Dialysis <2 years	162 (25.2)	481 (74.8)	643	**X^2 = 9.96; p=0.02**
Dialysis 2+ years	293 (30.0)	684 (70.0)	977	
Transplant	402 (29.4)	965 (70.6)	1367	
Age group†				
Less than 65	503 (24.7)	1537 (75.3)	2040	**X^2 = 19.86; p<0.0001**
65 and above	524 (31.3)	1151 (68.7)	1675	
Sex				
Male	635 (28.0)	1636 (72.0)	2271	X^2 = 0.01; p=0.92
Female	405 (27.8)	1054 (72.2)	1459	
Ethnicity‡				
White	902 (34.4)	1720 (65.6)	2622	**X^2 = 76.16; p<0.0001**
Black and minority ethnic	138 (17.9)	635 (82.1)	773	

† Age unknown for 15 patients; ‡ Ethnicity unknown for 335 patients; bold text indicates statistical significance

For peer review only

Table 2: Respondent characteristics and proportion by sub-group with mild to moderate distress

Characteristic	Respondents (%)	Mild to moderate distress (%)	Comparison of proportions (mild to moderate distress patients)†
All respondents	1040 (100.0)	346 (33.3)	
Study site			
Site 1	187 (18.0)	72 (38.5)	
Site 2	177 (17.0)	58 (32.8)	X^2 = 3.24; p=0.356
Site 3	323 (31.1)	107 (33.1)	
Site 4	353 (33.9)	109 (30.9)	
Stage on ESRD pathway			
Pre-RRT	182 (17.5)	64 (35.2)	
Dialysis <2 years	154 (14.8)	55 (35.7)	X^2 = 4.85; p=0.183
Dialysis 2+ years	300 (28.8)	109 (36.3)	
Transplant	404 (38.8)	118 (29.2)	
Dialysis type (n=454)			
Hospital/in-centre haemodialysis	343 (75.6)	129 (37.6)	
Home haemodialysis	31 (6.8)	13 (41.9)	X^2 = 3.36; p=0.186
Peritoneal dialysis	80 (17.6)	22 (27.5)	
Age group‡			
Less than 50	174 (16.9)	78 (44.8)	
50 to 69	441 (42.9)	145 (32.9)	**X^2 = 14.33; p=0.0008**
70 and above	414 (40.2)	119 (28.7)	
Sex			
Male	633 (60.9)	191 (30.2)	**X^2 = 6.63; p=0.01**
Female	407 (39.1)	155 (38.1)	
Ethnicity			
White	902 (86.7)	283 (31.4)	**X^2 = 10.36; p=0.0013**
Black and minority ethnic	138 (13.3)	63 (45.7)	

†Bold text denotes a statistically significant difference between groups; ‡Age unknown for four respondents

For peer review only

Table 3: Perceived need for help with distress by sub-group with mild to moderate distress (n=346)

Characteristic	Median 'need' score (IQR)	Patients scoring 4 or more on need thermometer (%)	Comparison of proportions (mild to moderate distress patients)†
All respondents	3 (1 to 5)	141 (40.8)	
Study site			
Site 1	1 (0 to 4)	55 (29.4)	
Site 2	1 (0 to 3)	38 (21.5)	**X^2 = 9.49; p=0.02**
Site 3	1 (0 to 3)	74 (22.9)	
Site 4	0 (0 to 3)	71 (20.1)	
Stage on ESRD pathway			
Pre-RRT	2 (0 to 4)	22 (34.9)	
Dialysis <2 years	3 (2 to 6)	35 (66.0)	**X^2 = 27.71; p<0.0001**
Dialysis 2+ years	4 (2 to 6)	59 (55.7)	
Transplant	2 (0 to 4)	35 (29.9)	
Dialysis type (n=454)			
Hospital/in-centre haemodialysis	4 (2 to 6)	70 (56.0)	
Home haemodialysis	4 (2 to 8)	7 (53.8)	X^2 = 5.44; p=0.07
Peritoneal dialysis	3 (0 to 5)	6 (28.6)	
Age group			
Less than 50	3 (2 to 5)	35 (44.9)	
50 to 69	3 (1 to 5)	55 (39.0)	X^2 = 0.85; p=0.654
70 and above	3 (1 to 5)	48 (41.4)	
Sex			
Male	3 (1 to 5)	73 (39.2)	X^2 = 0.54; p=0.462
Female	3 (1 to 5)	67 (43.8)	
Ethnicity			
White	3 (1 to 5)	106 (37.9)	**X^2 = 7.06; p=0.008**
Black and minority ethnic	5 (2 to 7)	34 (57.6)	

†Bold text indicates a statistically significant difference between groups

For peer review only

Table 4: Number and proportion of patients reporting problems on the DT problem list

**Problems by domain**	**Number of patients (%)†**	**Patients reporting at least 1 problem for the domain (%)**
Any problems (n=36)	-	339 (98.0)
Practical domain (n=5)		
Transport	84 (24.3)	
Insurance/financial	76 (22.0)	
Work	53 (15.3)	167 (48.3)
Housing	30 (8.7)	
Childcare	13 (3.8)	
Family domain (n=3)		
Dealing with friend/relative	66 (19.1)	
Dealing with partner	60 (17.3)	118 (34.1)
Dealing with children	27 (7.8)	
Emotional domain (n=6)		
Worry	257 (74.3)	
Loss of interest in usual activities	186 (53.8)	
Sadness	166 (48.0)	318 (91.9)
Depression	159 (46.0)	
Nervousness	150 (43.4)	
Fears	148 (42.8)	
Spiritual domain (n=1)		
Spiritual/religious concerns	23 (6.6)	-
Physical domain (n=21)		
Fatigue	248 (71.7)	
Skin dry/itchy	225 (65.0)	
Sleep	212 (61.3)	
Memory/concentration	183 (52.9)	
Pain	178 (51.4)	
Getting around	171 (49.4)	
Tingling in hands/feet	144 (41.6)	
Breathing	133 (38.4)	
Feeling swollen	127 (36.7)	
Bathing/dressing	119 (34.4)	329 (95.1)
Appearance	116 (33.5)	
Eating	111 (32.1)	
Nose dry/congested	108 (31.2)	
Constipation	106 (30.6)	
Nausea	100 (28.9)	
Changes in urination	94 (27.2)	
Indigestion	92 (26.6)	
Sexual	88 (25.4)	
Diarrhoea	79 (22.8)	
Mouth sores	42 (12.1)	
Fevers	31 (9.0)	

†Problems within each domain ranked from most to least frequently cited by respondents

For peer review only

Table 5: Median number of problems reported in each DT problem list domain, by patient group

**Characteristic**	**Practical†,‡**	**Family**	**Emotional**	**Physical**	**Total**
Stage on ESRD pathway	Median (IQR)§	Median (IQR)	Median (IQR)	Median (IQR)	Median (IQR)
Pre-RRT	1 (0 to 1)	0 (0 to 1)	3 (1 to 4)	**8 (4 to 12)**	**12 (7 to 17)**
Dialysis <2 years	1 (0 to 2)	0 (0 to 1)	3 (2 to 5)	**8 (6 to 12)**	**13 (9 to 17)**
Dialysis 2+ years	1 (0 to 1)	0 (0 to 1)	3 (2 to 5)	**9 (6 to 11)**	**13 (9 to 16)**
Transplant	0 (0 to 1)	0 (0 to 1)	3 (1 to 4)	**6 (3 to 10)**	**10 (5 to 16)**
				**p=0.019**	**p=0.023**
Dialysis type (n=164)					
Hospital/in-centre haemodialysis	1 (0 to 1)	0 (0 to 1)	3 (2 to 5)	8 (5 to 11)	13 (8 to 16)
Home haemodialysis	1 (0 to 2)	0 (0 to 1)	4 (2 to 5)	10 (8 to 13)	16 (11 to 19)
Peritoneal dialysis	0 (0 to 1)	0 (0 to 1)	3 (2 to 4)	8 (6 to 11)	12 (9 to 16)
Age group[4]					
Less than 50	**1 (0 to 2)**	**0 (0 to 1)**	**3 (2 to 5)**	7 (4 to 11)	**13 (8 to 17)**
50 to 69	**1 (0 to 2)**	**0 (0 to 1)**	**3 (2 to 5)**	8 (5 to 11)	**12 (8 to 18)**
70 and above	**0 (0 to 1)**	**0 (0 to 0)**	**3 (1 to 4)**	7 (4 to 10)	**11 (7 to 15)**
	**p<0.0001**	**p=0.001**	**p=0.038**		**p=0.033**
Sex					
Male	1 (0 to 1)	0 (0 to 1)	3 (2 to 4)	**7 (4 to 10)**	12 (7 to 16)
Female	0 (0 to 1)	0 (0 to 1)	3 (2 to 5)	**8 (5 to 12)**	12 (8 to 17)
				**p=0.043**	
Ethnicity					
White	0 (0 to 1)	0 (0 to 1)	3 (2 to 4)	8 (5 to 11)	12 (7 to 16)
Black and minority ethnic	0 (0 to 2)	0 (0 to 1)	4 (1 to 5)	7 (4 to 10)	11 (7 to 17)
All respondents (n=346)	0 (0 to 1)	0 (0 to 1)	3 (2 to 5)	8 (5 to 11)	12 (7 to 16)

†Bold text denotes a statistically significant difference between groups (Mann Whitney test for characteristics with two sub-groups; Kruskall Wallis test for characteristics with three or more sub-groups); ‡Spiritual domain excluded from analysis; §IQR = inter-quartile range

For peer review only

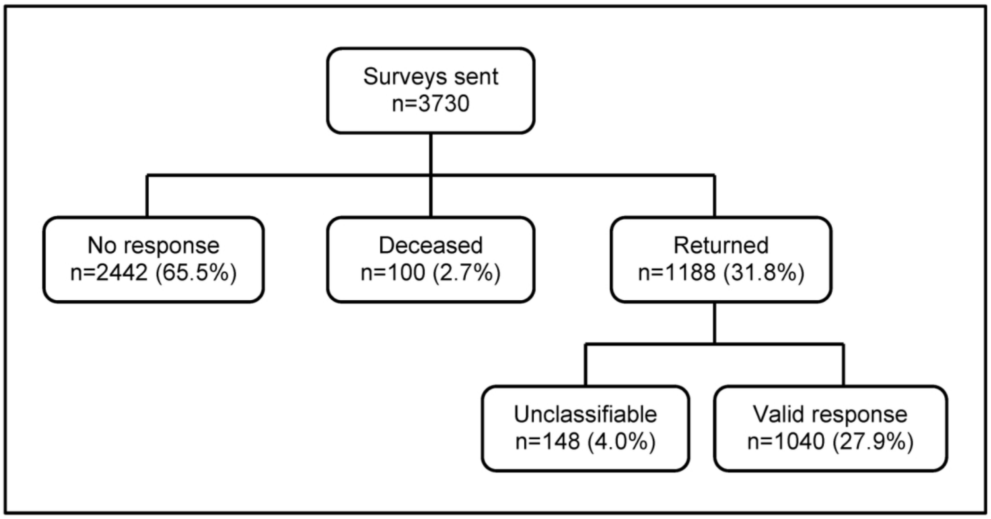

Figure 1: Surveys mailed and returned

150x78mm (300 x 300 DPI)

STROBE 2007 (v4) Statement—Checklist of items that should be included in reports of *cross-sectional studies*

Section/Topic	Item #	Recommendation	Reported on page #
**Title and abstract**	1	(*a*) Indicate the study's design with a commonly used term in the title or the abstract	1
		(*b*) Provide in the abstract an informative and balanced summary of what was done and what was found	2
**Introduction**			
Background/rationale	2	Explain the scientific background and rationale for the investigation being reported	4
Objectives	3	State specific objectives, including any prespecified hypotheses	4
**Methods**			
Study design	4	Present key elements of study design early in the paper	5-6
Setting	5	Describe the setting, locations, and relevant dates, including periods of recruitment, exposure, follow-up, and data collection	4-5
Participants	6	(*a*) Give the eligibility criteria, and the sources and methods of selection of participants	5
Variables	7	Clearly define all outcomes, exposures, predictors, potential confounders, and effect modifiers. Give diagnostic criteria, if applicable	5-6
Data sources/ measurement	8*	For each variable of interest, give sources of data and details of methods of assessment (measurement). Describe comparability of assessment methods if there is more than one group	5-6
Bias	9	Describe any efforts to address potential sources of bias	*5*
Study size	10	Explain how the study size was arrived at	6
Quantitative variables	11	Explain how quantitative variables were handled in the analyses. If applicable, describe which groupings were chosen and why	5-6
Statistical methods	12	(*a*) Describe all statistical methods, including those used to control for confounding	5-6
		(*b*) Describe any methods used to examine subgroups and interactions	5-6
		(*c*) Explain how missing data were addressed	6
		(*d*) If applicable, describe analytical methods taking account of sampling strategy	N/A
		(*e*) Describe any sensitivity analyses	N/A
**Results**			

For peer review only

Participants	13*	(a) Report numbers of individuals at each stage of study—eg numbers potentially eligible, examined for eligibility, confirmed eligible, included in the study, completing follow-up, and analysed	6; Fig 1
		(b) Give reasons for non-participation at each stage	N/A
		(c) Consider use of a flow diagram	Fig 1
Descriptive data	14*	(a) Give characteristics of study participants (eg demographic, clinical, social) and information on exposures and potential confounders	6
		(b) Indicate number of participants with missing data for each variable of interest	N/A
Outcome data	15*	Report numbers of outcome events or summary measures	6-7
Main results	16	(*a*) Give unadjusted estimates and, if applicable, confounder-adjusted estimates and their precision (eg, 95% confidence interval). Make clear which confounders were adjusted for and why they were included	6-7
		(*b*) Report category boundaries when continuous variables were categorized	6-7
		(*c*) If relevant, consider translating estimates of relative risk into absolute risk for a meaningful time period	N/A
Other analyses	17	Report other analyses done—eg analyses of subgroups and interactions, and sensitivity analyses	6-7
**Discussion**			
Key results	18	Summarise key results with reference to study objectives	8
Limitations	19	Discuss limitations of the study, taking into account sources of potential bias or imprecision. Discuss both direction and magnitude of any potential bias	9
Interpretation	20	Give a cautious overall interpretation of results considering objectives, limitations, multiplicity of analyses, results from similar studies, and other relevant evidence	8
Generalisability	21	Discuss the generalisability (external validity) of the study results	9-10
**Other information**			
Funding	22	Give the source of funding and the role of the funders for the present study and, if applicable, for the original study on which the present article is based	11

*Give information separately for cases and controls in case-control studies and, if applicable, for exposed and unexposed groups in cohort and cross-sectional studies.

Note: An Explanation and Elaboration article discusses each checklist item and gives methodological background and published examples of transparent reporting. The STROBE checklist is best used in conjunction with this article (freely available on the Web sites of PLoS Medicine at http://www.plosmedicine.org/, Annals of Internal Medicine at http://www.annals.org/, and Epidemiology at http://www.epidem.com/). Information on the STROBE Initiative is available at www.strobe-statement.org.

For peer review only
